# Supplementary figures and images for: The geometry of representational drift in natural and artificial neural networks
Source: PLoS Comput Biol. 2022 Nov 28;18(11):e1010716. doi: 10.1371/journal.pcbi.1010716 (PMC9731438; doi:10.1371/journal.pcbi.1010716)

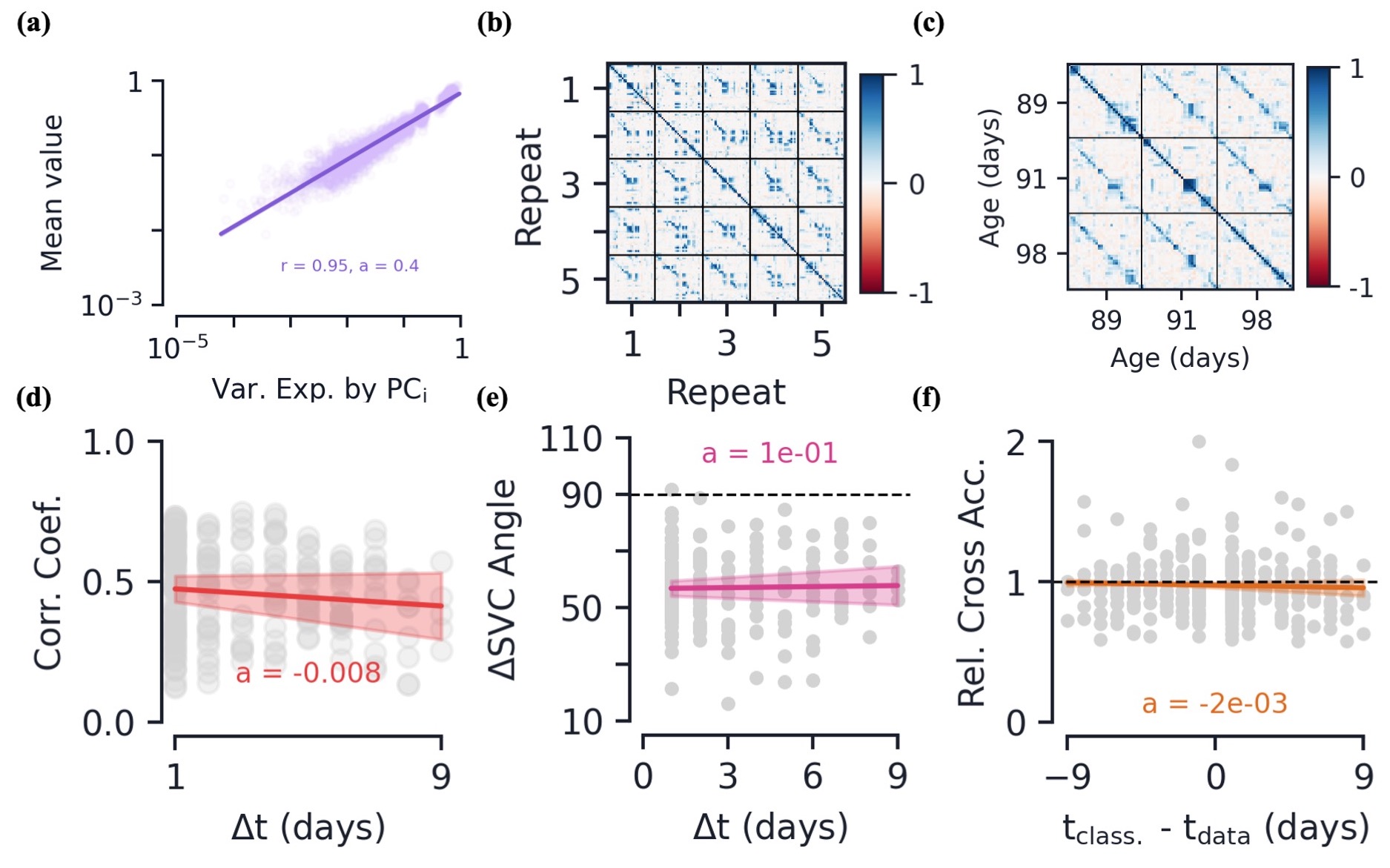

Supplement: S1 Fig — (a) Variance explained versus mean value along the corresponding PC direction, normalized by L2 magnitude of the mean response vector. Note all mean values are positive by definition of the PC’s direction. [b-d] Plots equivalent to Fig 5c, 5d and 5e, but instead of angle as a measure of similarity, here we are using Pearson’s correlation coefficient, Eq (7). These plots are provided to show a similarity of angle between response vectors (used in the main text) with the metric used in Ref. [11]. (b) Correlation within-session and across the first five movie repeats between response vectors. (c) Correlation between mean response vectors across the three session. (d) Correlation as a function of time between sessions, Δt. Again, we note this decrease is quite small as a function of time (< .01/day). [e-f] SVC metrics as a function of time difference between sessions. These plots are analogous to Fig 3e and 3f, just plotted as a function of Δt instead of by session number. Once again, note both of these metrics are relatively stable as a function of the time difference. (e) Angle between SVC classifiers. (f) Relative cross classsification accuracy. Note the time difference here can go negative beause the classifier could be trained on a sesssion earlier than the data it is tested on (and unlike the angle between classifiers, in general arel.(−t) ≠ arel.(t)). (JPG) [file pcbi.1010716.s001.jpg]

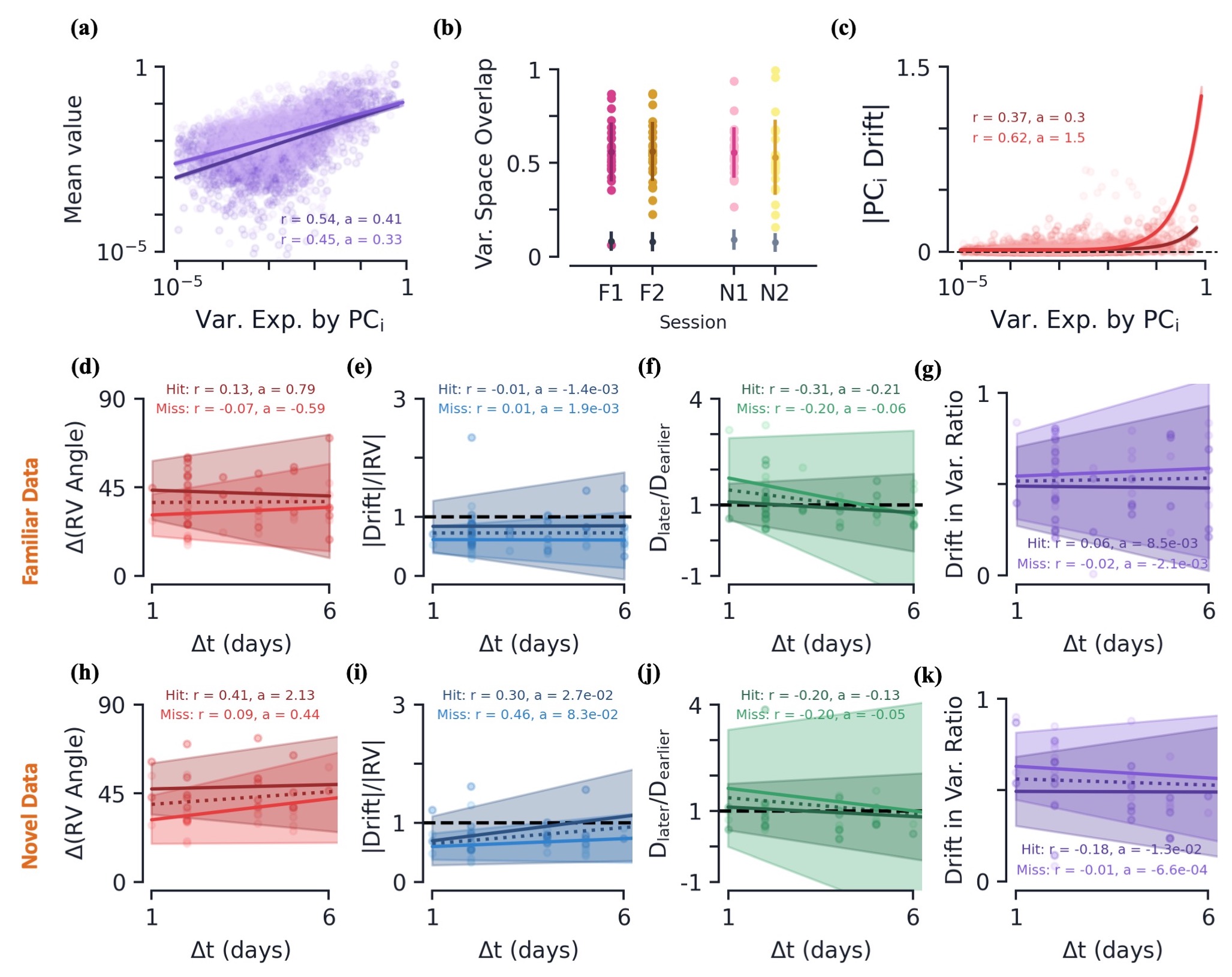

Supplement: S2 Fig — (a) Variance explained versus mean value along corresponding PC direction, normalized by L2 magnitude of mean response vector. Note all mean values are positive by definition of the PC’s direction. (b) Variational space overlap, Γ of Eq (10), between the Hit and Miss stimulus groups for each session. Pink/yellow light dots show raw data, pink/yellow dark dots/lines show average ± s.e. Grey dots/lines show average ± s.e. of two randomly oriented variational spaces of same dimensions. Notably, the two stimulus groups overlap significantly more than chance. (c) Magnitude of drift as a function of the earlier session’s variance explained, but unlike Fig 4c the magnitude is not normalized by the full L2 magnitude of the drift. This plot is meant to show the comparatively larger drift of the novel sessions (darker dots/lines) relative to the familiar sessions (lighter dots/lines). [d-k] Various metrics as a function of the time between earlier and later session, Δt, for both Hit and Miss trials, across all mice. The middle and bottom rows correspond to the familiar and novel data, respectively. Colored curves are linear regression fits with the shaded region showing all fits within the 95% confidence intervals of the slope and intercept. Darker/lighter colored lines/points correspond to Hit and Miss trials, respectively. Dotted lines are best fits for class-averaged data. (d, h) Average angle between response vectors (Methods). (e, i) Average magnitude of drift relative to magnitude of mean response vector. (f, j) Average ratio of participation ratio from later session to earlier session. Note this differs from the analogous plot for the passive and ANN data in that we are taking the ratio rather than the difference. We found ratio to be a better measure given the significant variance in size of variational/neural state spaces for the behavioral data. (g, k) Average drift magnitude within earlier session’s variational space, ratio relative to full drift magnitude. (JPG) [file pcbi.1010716.s002.jpg]

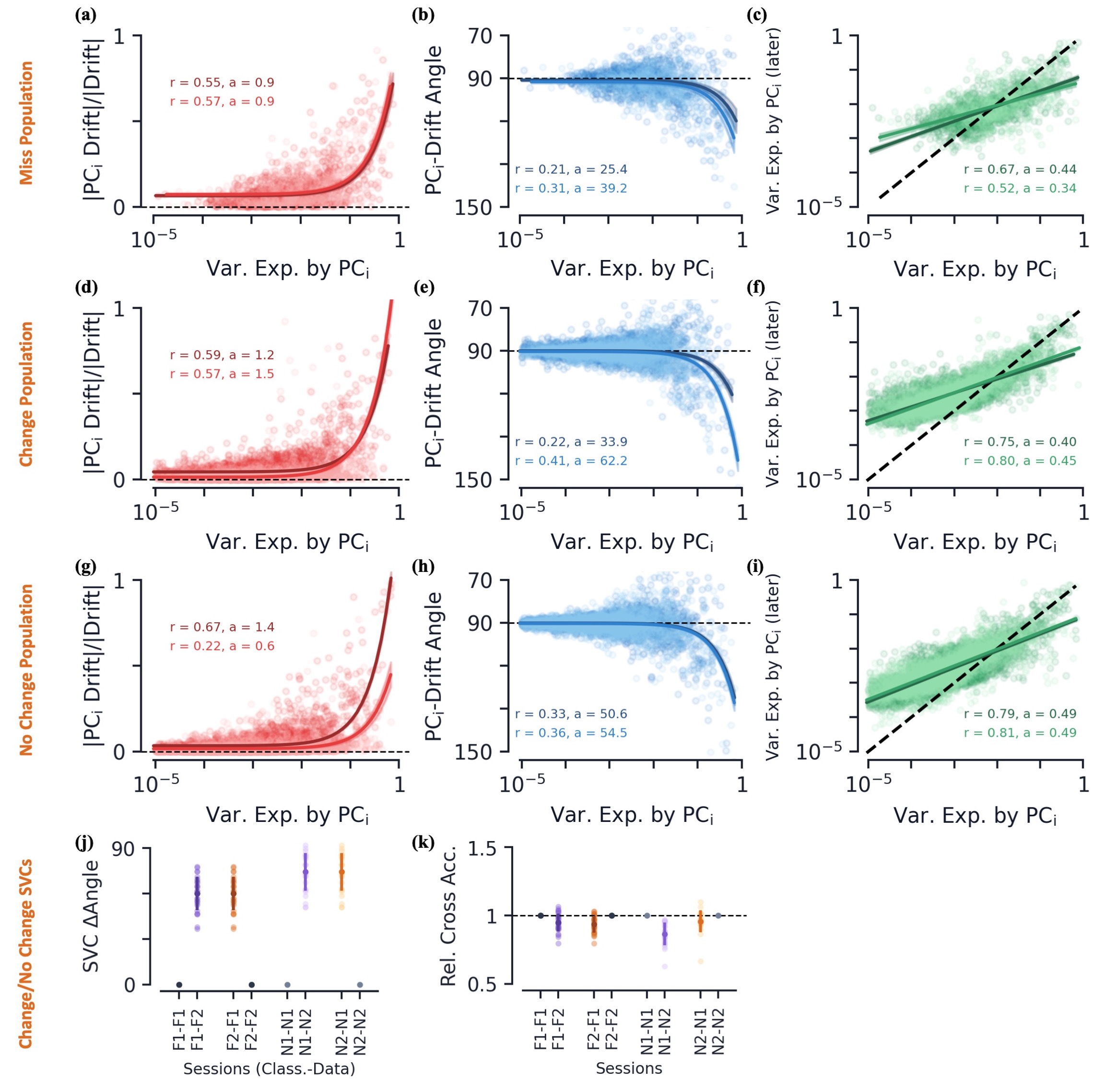

Supplement: S3 Fig — [a-i] Various drift metrics of Miss (first row), Change (second row), and No Change (third row) trials and their dependence on PC dimension of the earlier session’s variational space. Dark colors correspond to drift between familiar sessions, while lighter colors are those between novel sessions. Metrics are plotted as a function of the stimulus group PC’s ratio of variance explained, vi. Colored curves are linear regression fits. (a, d, g) Magnitude of drift along PC direction relative to full (L2) magnitude of drift. (b, e, h) Angle of drift with respect to PC direction. (c, f, i) Post-drift variance explained along PC direction (dotted line is equality). Linear regression fit to log(var. exp). [j-k] Various SVC metrics as a function of session(s) for the Change/No Change stimulus groups. Dark solid dots/lines show average values with ± s.e. Light colored dots/lines show raw mice data. (j) Angle between SVC normal vectors. (k) Relative cross classification accuracy, Eq (14), as a function of test data session and trained data session. (JPG) [file pcbi.1010716.s003.jpg]

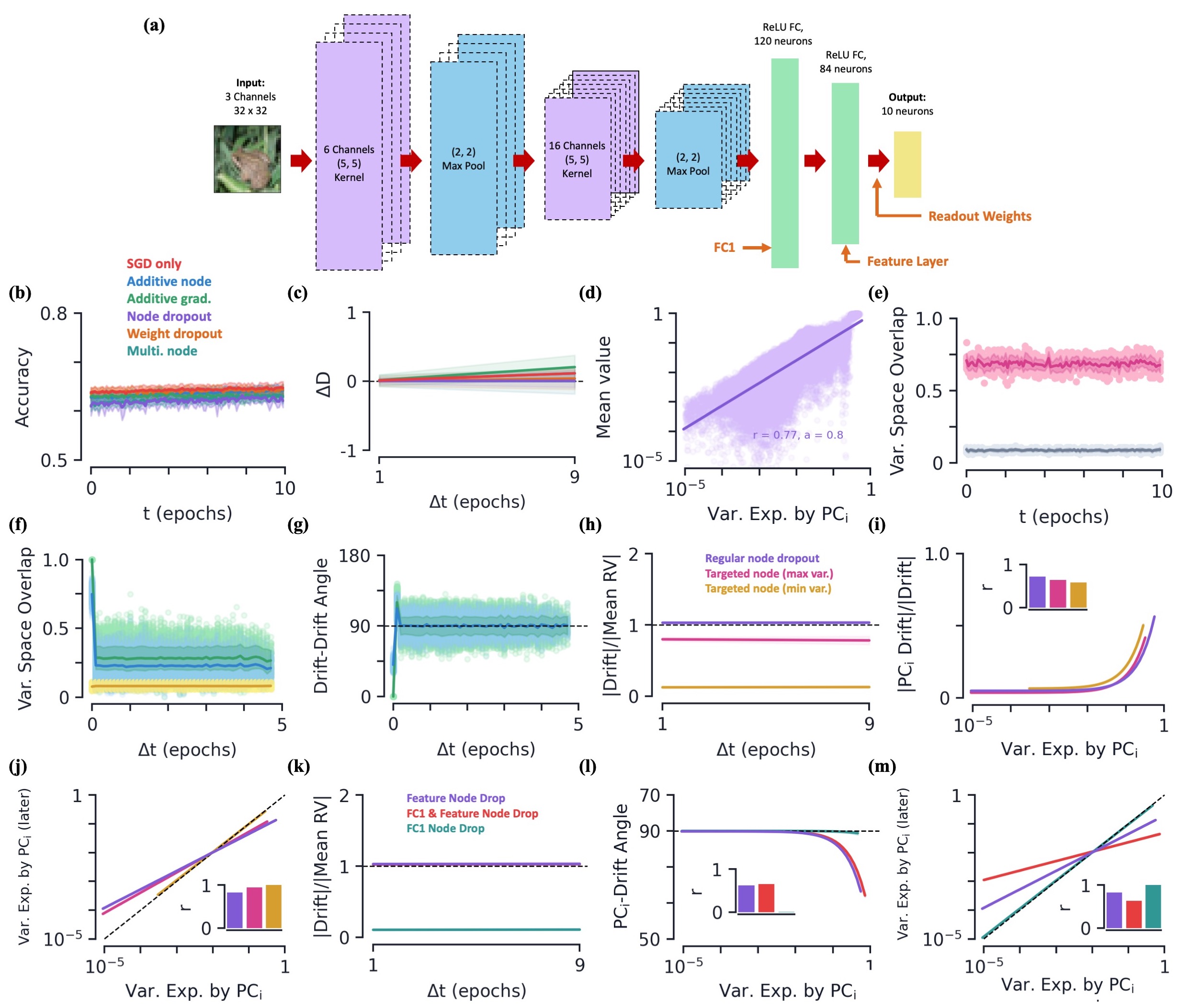

Supplement: S4 Fig — (a) Visualization of convolutional neural network architecture. (b) Accuracy as a function of epoch for SGD and all five types of noise. Note, for each of the six plots, t = 0 is chosen to be the point where the network has relatively steady accuracy, and does not necessarily correspond to the same epoch across noise types. (c) Average change in the variational space dimension, D, from later session to earlier session as a function of Δt. [d-g] Additional results for node dropout, with p = 0.5. (d) Variance explained versus mean value along corresponding PC direction, normalized by L2 magnitude of mean response vector. (e) Variational space overlap, Γ of Eq (10), between the two stimulus groups (frogs and birds) as a function of time (after steady accuracy is achieved) for node dropout with p = 0.5. Light pink dots show raw data for each trial, dark line/fill is mean ± s.e. Grey dots/lines show raw data and mean ± s.e. of two randomly oriented variational spaces of same dimensions. (f) Variational space overlap as a function of time difference. Green shows Γ between same stimulus group, blue shows between two different stimulus groups, yellow shows chance percentage. Between the two classes, overlap is largest for Δt = 0, before reaching a steady value that is still larger than chance. (g) Angle between drift vectors as a function of time difference between initial sessions. Green shows between same stimulus group, blue shows between two different groups. [h-j] Various plots for targeted node dropout. Regular node dropout, with p = 0.5, is plotted in purple. Maximum and minimum variance targeted node dropout, Eqs (21) and (22), are plotted in pink and yellow, respectively. (h) Average magnitude of drift relative to magnitude of mean response vector as a function of Δt. (i) As a function of the variance explained of the earlier session, magnitude of drift along corresponding PC direction, normalized by full (L2) magnitude of drift. (j) Post-drift variance explained a [file pcbi.1010716.s004.jpg]

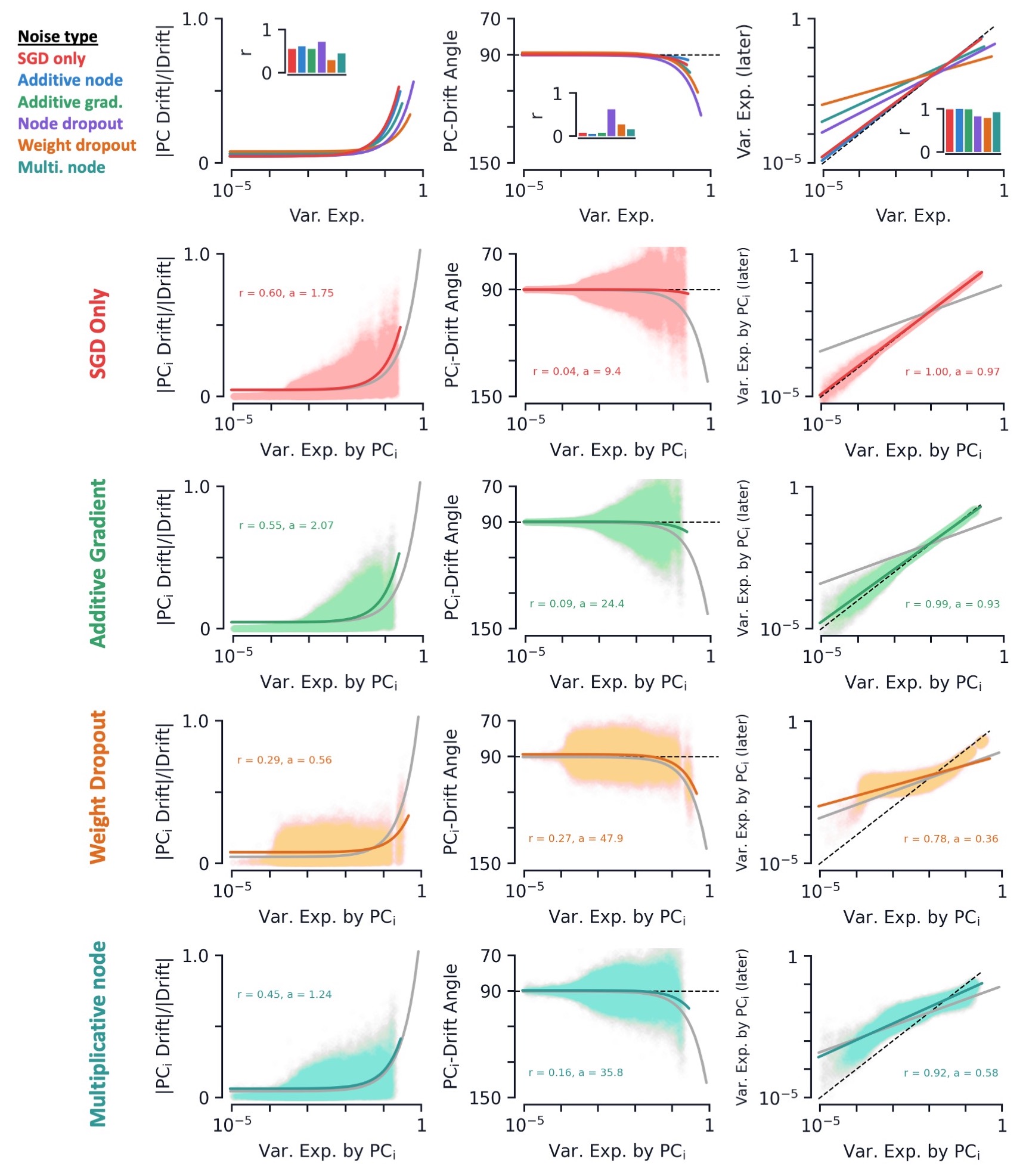

Supplement: S5 Fig — Various drift metrics and their dependence on PC dimension of the earlier session’s variational space. Metrics are plotted as a function of stimulus group PC’s ratio of variance explained, vi. Colored curves are linear regression fits. Grey curves are behavioral data fits from the novel sessions shown in Fig 4c, 4d and 4e. Noise hyperparameters are chosen to be best fits to experimental data across hyperparameter scans, see Fig 5a. Note equivalent plots for additive node and node dropout are in Fig 5 of the main text. (First column) Magnitude of drift along PC direction relative to full (L2) magnitude of drift. (Second column) Angle of drift with respect to PC direction. (Third column) Post-drift variance explained along PC direction (dotted line is equality). Linear regression fit to log(var. exp.). (First row) Fits for only SGD and all five types of noise. Insets show r-values of fits. (Second row) Only SGD. (Third row) Additive gradient noise with σ = 3.0. (Fourth row) Weight dropout with p = 0.6. (Fifth row) Multiplicative node with σ = 1.0. (JPG) [file pcbi.1010716.s005.jpg]

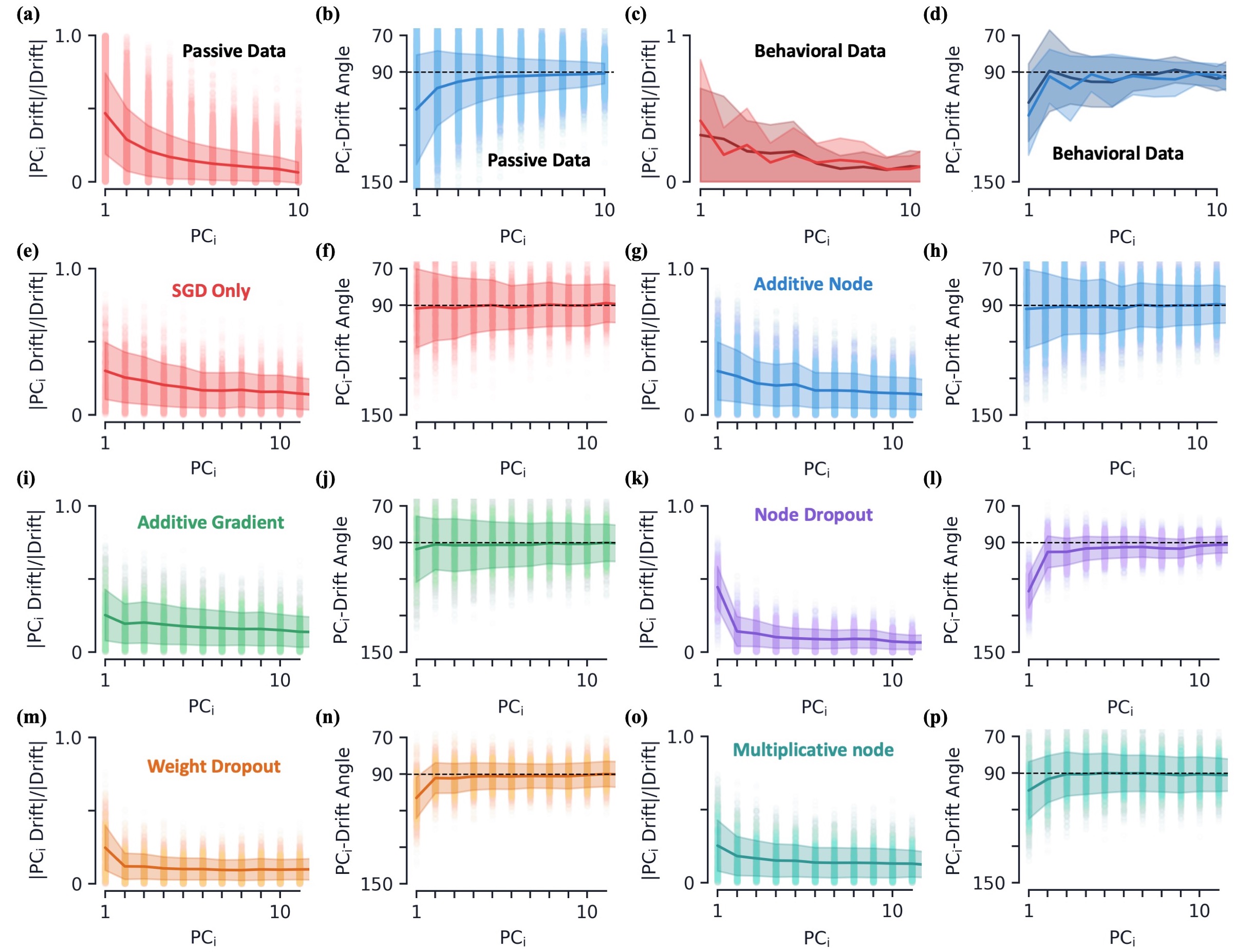

Supplement: S6 Fig — Various drift metrics and their dependence on PC dimension of the earlier session, rather than its’s variational space, which is used throughout the main text. Solid dark line and shading represent mean ± s.e. for each PC. Raw data is scattered behind as points. (First and third column) Magnitude of drift along PC direction relative to full (L2) magnitude of drift. (Second and fourth column) Angle of drift with respect to PC direction. Noise hyperparameters are chosen to be best fits to experimental data across hyperparameter scans, see Fig 5a. [a-b] Passive data. [c-d] Behavioral Hit data, with darker/lighter lines/shading the familiar and novel sessions, respectively. Note raw data is not scattered for clarity. [e-f] ANN with only noise due to SGD. [g-h] Additive node with σ = 0.1. [i-j] Additive gradient with σ = 3.0. [k-l] Node dropout with p = 0.5. [m-n] Weight dropout with p = 0.6. [o-p] Multiplicative node with σ = 1.0. (JPG) [file pcbi.1010716.s006.jpg]

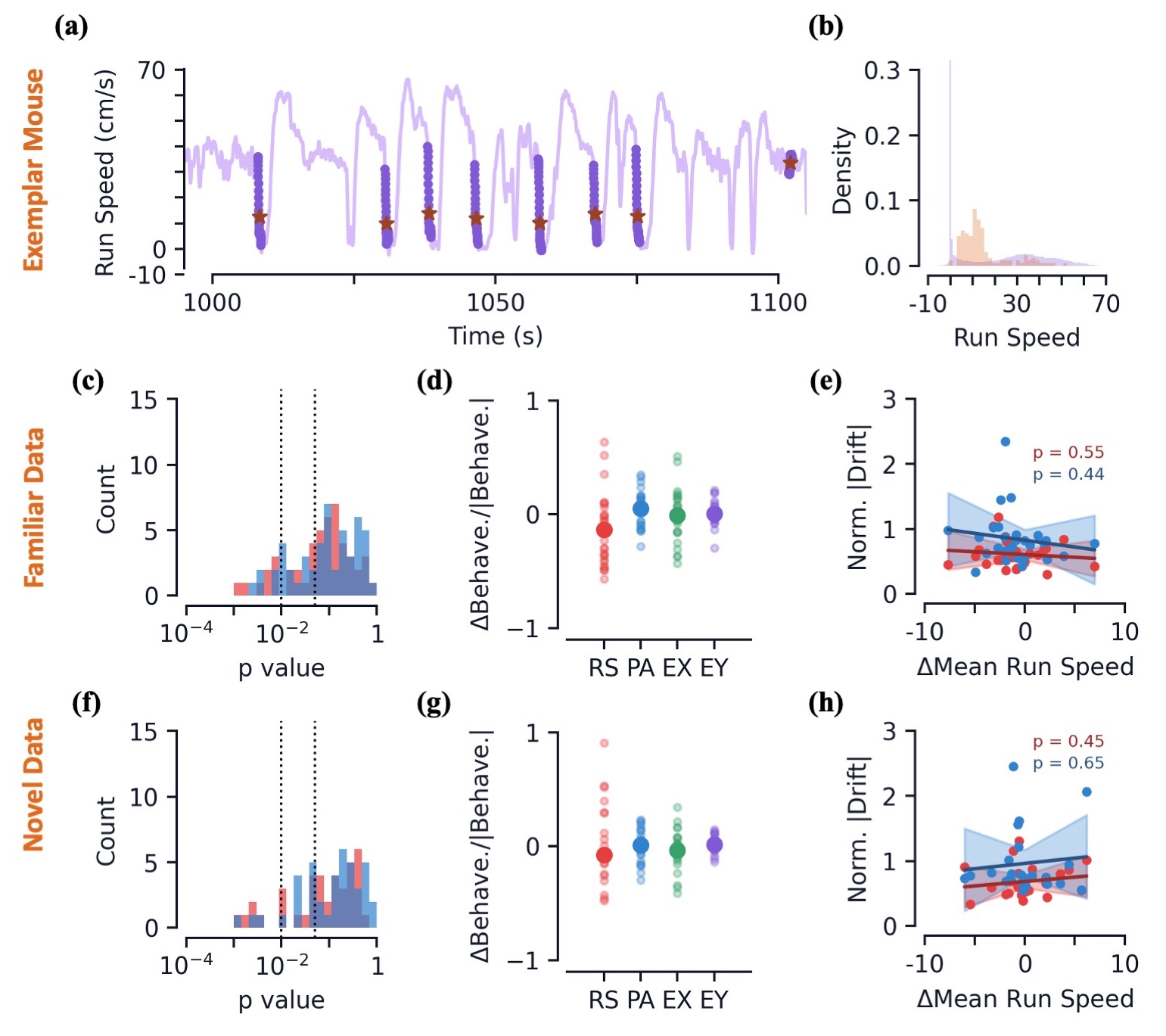

Supplement: S7 Fig — [a,b] Behavior data from an exemplar mouse (specifically from a familiar session). (a) Run speed versus time for a sample time slice. Light purple shows raw data, dark purple points shows data during engaged Hit or Miss trials, and orange stars show average run speed for each Hit or Miss trial. (b) Distribution of run speeds over entire session. Purple shows raw data, orange shows average over each Hit or Miss trial. [c-e] Familiar sessions behavior control. (c) Histogram of p-values from F-test to see if the four behavioral metrics (run speed, pupil area, x and y eye position) could explain size of response vectors, across all mice used in familiar data. Red and blue shown Hit and Miss stimulus groups, respectively. Two dotted vertical lines mark the 0.01 and 0.05 significance levels. These are the p-values for individual tests, such that one out of 20 (100) are expected to below 0.05 (0.01) by chance. (d) Normalized behavioral differences, see Eq (16), between F1 and F2 sessions for run speed (RS), pupil area (PA), and x and y eye position (EX, EY). Large dots are means across all mice, with individual mice data scatter behind. (e) Scatter plots of normalized drift magnitude, ‖d‖2/n, as a function of change in mean run speed for mice used in familiar data. Dark lines show linear fits to data and respective p-values of Wald tests to see if slopes are significantly different from flat are shown. Red and blue shown Hit and Miss stimulus groups, respectively. [f-h] Novel sessions behavior control. (f) Same as (c), for novel sessions. (g) Same as (d), for N1 and N2 sessions. (h) Same as (e), for novel sessions. (JPG) [file pcbi.1010716.s007.jpg]

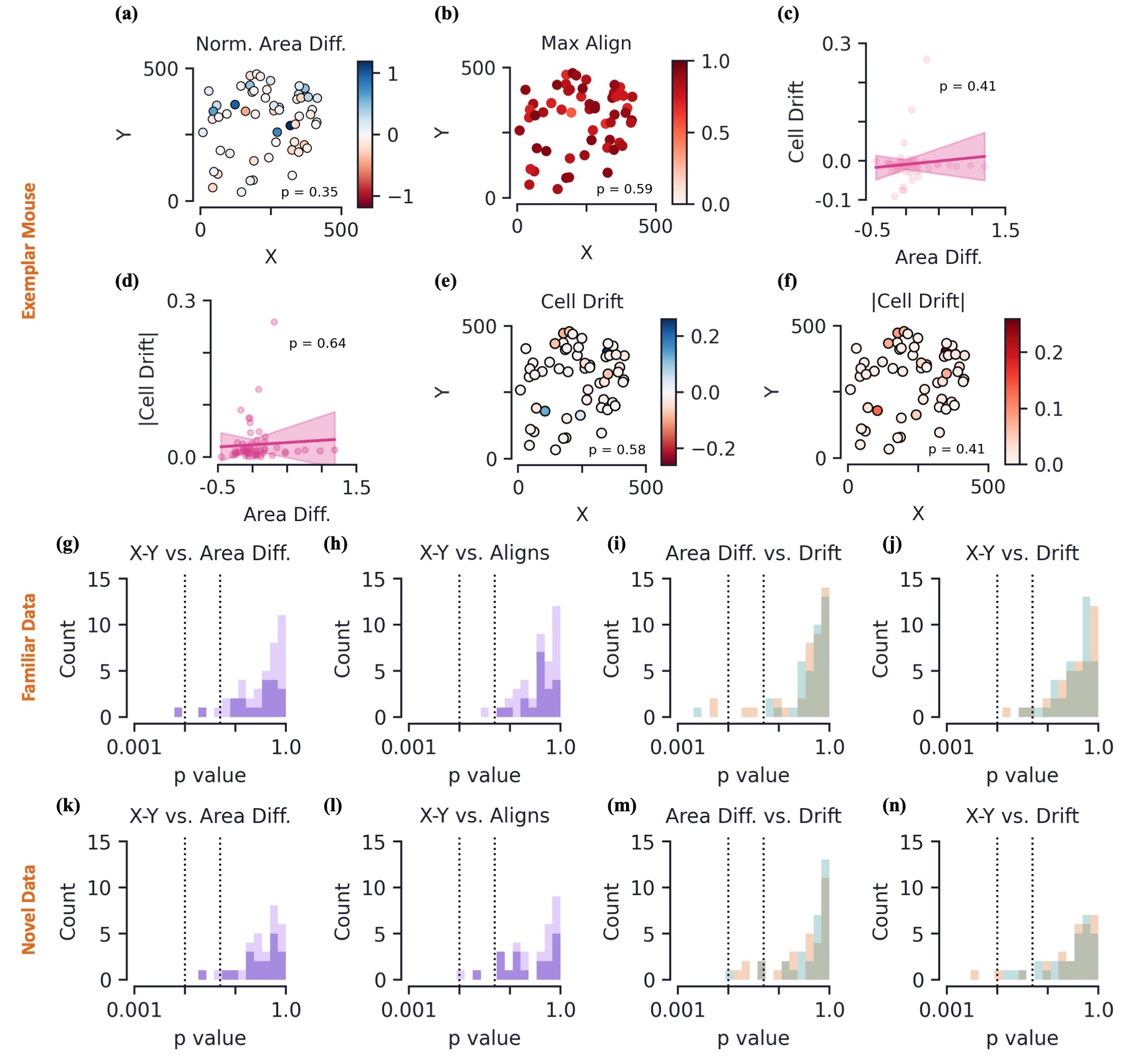

Supplement: S8 Fig — [a-f] Results from an exemplar mouse’s drift (specifically from novel Hit population drift when relevant). (a) Normalized area difference of ROI masks, Eq (17), as a function of the later session’s X-Y locations in the imaging plane. P-value shown is from F-test to see if planar fit to data is significantly different from flat. (b) Maximum alignment between ROI masks, Eq (18), as a function of the later session’s X-Y location. P-value from same type of test used in (a). (c) Scatter plot of each cell’s drift as a function of the cell’s normalized area difference. Dark line is a linear fit with shaded area representing mean ±2 s.e., p-value from Wald Test to see if slope of fit is significantly different from flat. (d) Same as (c), but magnitude of a cell’s drift. (e) Each cell’s drift as a function of the later session’s X-Y locations. P-value from same type of test used in (a). (f) Same as (e), but magnitude of a cell’s drift. [g-j] Histogram of p-values of the same tests shown in (a), (b), (c), and (e), respectively, across all mice used in familiar data. Two dotted vertical lines mark the 0.01 and 0.05 significance levels. These are the p-values for individual tests, such that one out of 20 (100) are expected to below 0.05 (0.01) by chance. (g, h) Dark values represent data from single-plane imaging while lighter bars are individual imaging planes from multi-plane data (data is stacked). (i, j) Orange and teal bar plots (not stacked) represent data from the Hit and Miss population drifts, respectively. [k-n] Same as [g-j], but for the all mice used in the novel data. (JPG) [file pcbi.1010716.s008.jpg]

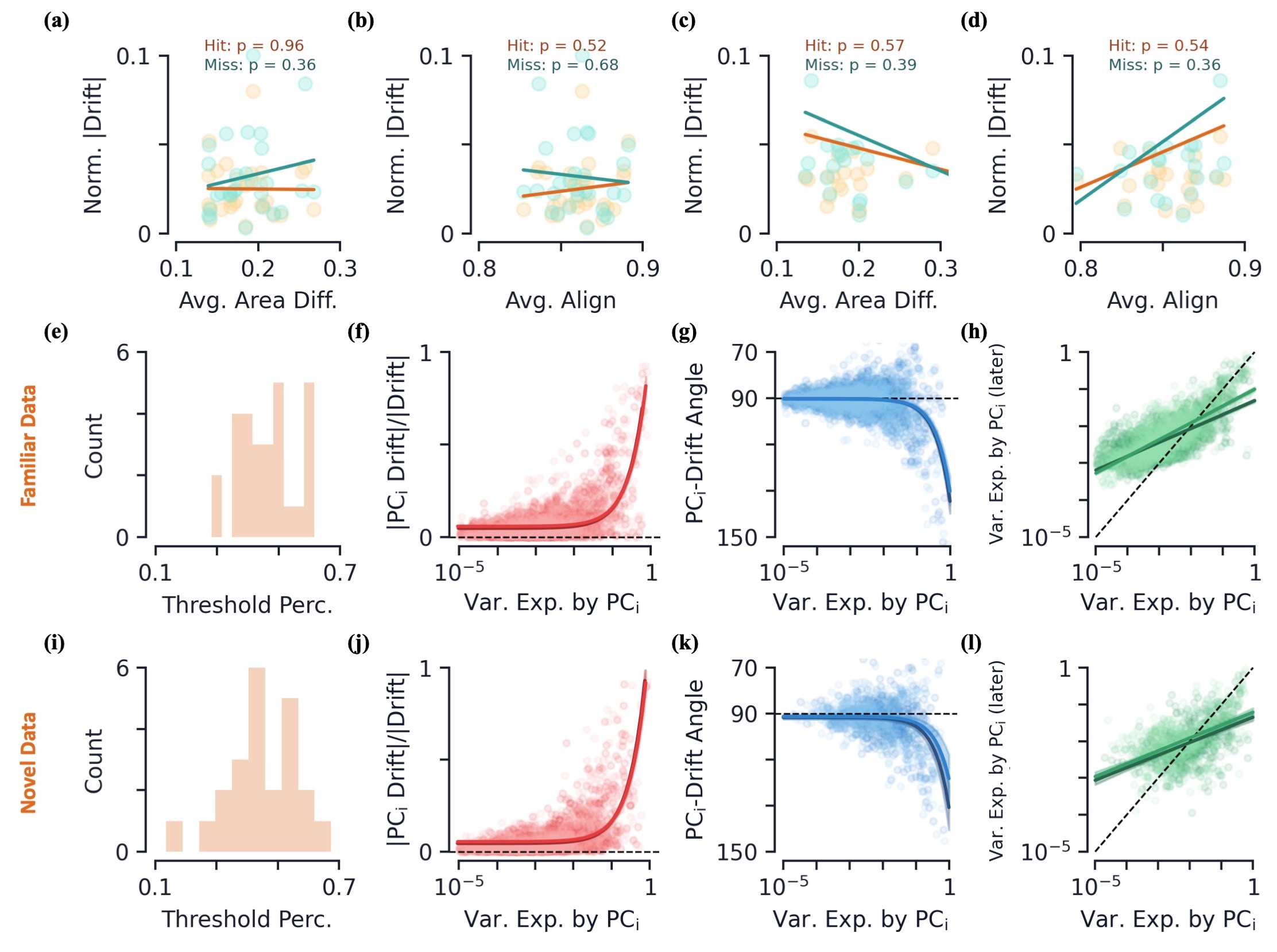

Supplement: S9 Fig — [a-f] Results from an exemplar mouse’s drift (specifically from novel Hit population drift when relevant). (a) Normalized area difference of ROI masks, Eq (17), as a function of the later session’s X-Y locations in the imaging plane. P-value shown is from F-test to see if planar fit to data is significantly different from flat. (b) Maximum alignment between ROI masks, Eq (18), as a function of the later session’s X-Y location. P-value from same type of test used in (a). (c) Scatter plot of each cell’s drift as a function of the cell’s normalized area difference. Dark line is a linear fit with shaded area representing mean ±2 s.e., p-value from Wald Test to see if slope of fit is significantly different from flat. (d) Same as (c), but magnitude of a cell’s drift. (e) Each cell’s drift as a function of the later session’s X-Y locations. P-value from same type of test used in (a). (f) Same as (e), but magnitude of a cell’s drift. [g-j] Histogram of p-values of the same tests shown in (a), (b), (c), and (e), respectively, across all mice used in familiar data. Two dotted vertical lines mark the 0.01 and 0.05 significance levels. These are the p-values for individual tests, such that one out of 20 (100) are expected to below 0.05 (0.01) by chance. (g, h) Dark values represent data from single-plane imaging while lighter bars are individual imaging planes from multi-plane data (data is stacked). (i, j) Orange and teal bar plots (not stacked) represent data from the Hit and Miss population drifts, respectively. [k-n] Same as [g-j], but for the all mice used in the novel data. (JPG) [file pcbi.1010716.s009.jpg]
