# Supplementary material for: The geometry of representational drift in natural and artificial neural networks
Source: PLoS Comput Biol. 2022 Nov 28;18(11):e1010716. doi: 10.1371/journal.pcbi.1010716 (PMC9731438; doi:10.1371/journal.pcbi.1010716)
Supplement: S1 Appendix — Here we argue that Γps,s′, defined in Eq (10), is invariant with respect to rotations of the orthonormal bases used to span the variational spaces of s and s′. (PDF) [file pcbi.1010716.s010.pdf]

S1 Appendix.

Variational space overlap rotational invariance. Here we argue that $\Gamma_p^{s,s'}$, defined in Eq (10), is invariant with respect to rotations of the orthonormal bases used to span the variational spaces of s and s'. For brevity, we drop all p subscripts here. To show this, without loss of generality, we move to the basis where $[\mathbf{w}_i^s]_\mu \equiv w_{i\mu}^s = \delta_{i\mu}$ where $\delta_{i\mu}$ is the Kronecker delta function, $\mu = 1, \ldots, n$, and $[\mathbf{w}_i^s]_\mu$ is the μth element of the vector $\mathbf{w}$, which we write as $w_{i\mu}^s$. Without any rotation, we have

$$\begin{aligned}\Gamma^{s,s'} &= \frac{1}{\min(\lceil D^s \rceil, \lceil D^{s'} \rceil)} \sum_{i=1}^{\lceil D^s \rceil} \sum_{j=1}^{\lceil D^{s'} \rceil} \left(\sum_{\mu=1}^{n} w_{i\mu}^s w_{j\mu}^{s'} \right)^2 , \\ &= \frac{1}{\min(\lceil D^s \rceil, \lceil D^{s'} \rceil)} \sum_{i=1}^{\lceil D^s \rceil} \sum_{j=1}^{\lceil D^{s'} \rceil} \left(w_{ji}^{s'} \right)^2 , \end{aligned} \quad (1)$$

where in the second line we have evaluated the sum over the Kronecker delta.

With our choice of basis, any rotation to another orthonomal basis of the $\lceil D^s \rceil$-dimensional subspace can be written in block diagonal form

$$\mathbf{R} = \begin{bmatrix} \mathbf{R}' & 0 \\ 0 & \mathbf{1} \end{bmatrix} , \quad (2)$$

where $\mathbf{R} \in \mathbb{R}^{n \times n}$, $\mathbf{R}' \in \mathbb{R}^{\lceil D^s \rceil \times \lceil D^s \rceil}$ is an orthogonal matrix, and $\mathbf{1}$ is the identity matrix. Let the elements of $\mathbf{R}$ be $r_{\mu\nu}$ for $\mu, \nu = 1, \ldots, n$ and thus the elements of $\mathbf{R}'$ are r_{ij} for $i, j = 1, \ldots, \lceil D^s \rceil$ Since $\mathbf{R}'$ is orthogonal, it obeys $\mathbf{R}'\mathbf{R}'^T = \mathbf{1}$, or in terms of its elements, $\sum_{j=1}^{\lceil D^s \rceil} r_{ij} r_{kj} = \delta_{ik}$. Now consider $\Gamma^{s,s'}$ after we have applied the basis transformation,

$$\begin{aligned}\Gamma^{s,s'} &= \frac{1}{\min(\lceil D^s \rceil, \lceil D^{s'} \rceil)} \sum_{i=1}^{\lceil D^s \rceil} \sum_{j=1}^{\lceil D^{s'} \rceil} \left(\mathbf{R}\mathbf{w}_i^s \cdot \mathbf{w}_j^{s'} \right)^2 , \\ &= \frac{1}{\min(\lceil D^s \rceil, \lceil D^{s'} \rceil)} \sum_{i=1}^{\lceil D^s \rceil} \sum_{j=1}^{\lceil D^{s'} \rceil} \left(\sum_{\mu,\nu=1}^{N} r_{\mu\nu} w_{i\nu}^s w_{j\mu}^{s'} \right)^2 , \\ &= \frac{1}{\min(\lceil D^s \rceil, \lceil D^{s'} \rceil)} \sum_{i=1}^{\lceil D^s \rceil} \sum_{j=1}^{\lceil D^{s'} \rceil} \left(\sum_{\mu=1}^{N} r_{\mu i} w_{j\mu}^{s'} \right)^2 , \end{aligned} \quad (3)$$

where in the last line we have again evaluated the sum over the Kronecker delta. Now use the fact that the elements of the rotation matrix, $r_{\mu i}$, are only nonzero for $\mu = 1, \ldots, \lceil D^s \rceil$, since it is block diagonal. Thus, without loss of generality we can write

the sum over μ into a sum over $k = 1, \ldots, \lceil D^s \rceil$,

$$\begin{aligned}
\Gamma^{s,s'} &= \frac{1}{\min(\lceil D^s \rceil, \lceil D^{s'} \rceil)} \sum_{i=1}^{\lceil D^s \rceil} \sum_{j=1}^{\lceil D^{s'} \rceil} \left(\sum_{k=1}^{\lceil D^s \rceil} r_{ki} w_{jk}^{s'} \right)^2 , \\
&= \frac{1}{\min(\lceil D^s \rceil, \lceil D^{s'} \rceil)} \sum_{j=1}^{\lceil D^{s'} \rceil} \sum_{i,k,m=1}^{\lceil D^s \rceil} r_{ki} w_{jk}^{s'} r_{mi} w_{jm}^{s'} , \\
&= \frac{1}{\min(\lceil D^s \rceil, \lceil D^{s'} \rceil)} \sum_{j=1}^{\lceil D^{s'} \rceil} \sum_{k,m=1}^{\lceil D^s \rceil} \delta_{km} w_{jk}^{s'} w_{jm}^{s'} , \\
&= \frac{1}{\min(\lceil D^s \rceil, \lceil D^{s'} \rceil)} \sum_{j=1}^{\lceil D^{s'} \rceil} \sum_{k=1}^{\lceil D^s \rceil} \left(w_{jk}^{s'} \right)^2 , \qquad (4)
\end{aligned}$$

where in the third line we have used the relation between $\mathbf{R}'$ above. Up to summation indices, this result is identical to Eq (1). Thus, $\Gamma^{s,s'}$ is invariant under rotations of the orthonormal basis of s, and since $\Gamma^{s,s'}$ is symmetric with respect to interchange of s and s', invariance under the same types of rotations of s' immediately follows.
