# Supplementary material for: The geometry of representational drift in natural and artificial neural networks
Source: PLoS Comput Biol. 2022 Nov 28;18(11):e1010716. doi: 10.1371/journal.pcbi.1010716 (PMC9731438; doi:10.1371/journal.pcbi.1010716)
Supplement: Attachment — Submitted filename: Aitken_review_2022.pdf [file pcbi.1010716.s011.pdf]

Authors define geometry of neuronal representations to better understand how they drift. Authors find that neural representations change the most in dimensions with highest variance. This geometry of drift in the visual cortex appears to be similar to drift in ANNs with dropout. The authors conclude with a hypothesis that drift could help prevent overfitting. It is interesting that the rate of drift was similar across familiar and novel stimulus presentations.

Representational drift is a feature of neural data that was recently reported in the hippocampus and cerebral cortex. This feature must be quantified and characterized in order to better develop an understanding for potential roles in neural computation. Therefore, the goals of this work are interesting and important. However, authors' methods of characterization are limited in providing further insight beyond what has already been reported. Importantly, there's little evidence for representational drift happening at all in this work. More careful comparison across sessions 1 and 2 compared to sessions 1 and 3 will be crucial to verify that representational drift is measurable in this data. Additionally, the contribution of changes in behavior across sessions was not addressed. While previous work has shown that behavioral confounds don't explain all neural change associated with drift, it is important to examine the contribution of behavioral change in any careful analysis of neural data. The dataset analyzed in this work contains behavioral information that could be analyzed for a revised draft. Extensive work must be done to demonstrate accumulation of change over time associated with drift, and also robustness controls for the mean response vector. These revisions are possible given the previously collected data; therefore, major revisions should be required before accepting this work for publication.

Major Concerns:

*** Based on previous work, we might expect the magnitude of drift to increase as the gap between sessions increases. I couldn't find evidence of this anywhere in the paper beyond an example figure in 2c and d. In line 168, authors state that an SVC can distinguish separate sessions. Does decoder performance improve with a larger session gap? (1-3 should have higher performance than 1-2 or 2-3)? This does not appear to be the case (or is very weakly present) in Figure 2e across different stimulus groups. How does the same plot look for the same stimulus groups (summary figures for 2c and d)? How does the rate of change in 2e compare to previous studies (ie. Xia J, Marks TD, Goard MJ, et al. (2021) etc)? More direct comparisons to previous work would be helpful.

**The authors seem to have missed an important line of work that is highly relevant to their findings from Denise Cai's group. This work shows that there is an 'excitable pool'

of cells that varies over days. The neurons that are most active are part of the excitable pool on any given day. As the excitable pool changes across days, neurons that were most active, are no longer active on subsequent days. These findings would result in a phenomenon similar to dropout. Authors should reference this work extensively throughout (Cai 2016, Mau 2020, etc).

** Importantly, behavioral measures were not included at all in this entire work. The available datasets include metrics like running speed, eye position and pupil area. These variables should be analyzed as extensively as the neural data and included in all session comparisons. For example, in all decoding analyses, is it possible to decode which session a dataset came from based on running speed alone?

* Line 170 : Authors cite representational drift work in hippocampus [11] as not being linked to behavioral features and claim that this is reasoning to not analyze such data in visual cortex. This explanation is not sufficient, especially given several works citing the relevance of behavioral features when analyzing neural data (Musall et al 2019, Clopath et al 2017, Liberti 2022).

All comparisons between odd and even trials should also include a comparison to the first and second half of sessions in order to determine whether there was any drift recorded over a single session (ex. in line 169 and 282). Additionally 3e,f should have odd even/ first half second have same day comparisons.

Line 9: The idea that neurons represent external stimuli is a hypothesis and should be stated as such. (Brette, 2018; Freeman and Skarda, 1990) The authors talk a lot about representations but make no effort to link neural activity to any particular representation. Given (Musall et al 2019) it will be important to determine which external stimuli and behavioral variables are contributing to neuronal activity.

Line 248: Authors state that decoding accuracies are higher than in previous work [8,9]. However, my understanding is that the timescale for this work is much shorter and it is particularly difficult to compare results to [9], where authors performed an 8-way classification. What is the actual accuracy? The results in the present study are obfuscated by plotting relative accuracy. A more direct comparison to previous work would be more informative.

Authors claim that the number of days between sessions is only weakly correlated with drift starting at line 179. I don't see any comparison between the number of days between sessions compared to the number of sessions, as was reported in [9]. The

authors should do a more thorough analysis as in figure 4d of ref [9] before making these claims.

When reporting low dimensional subspaces, authors should cite other work that has reported similar properties (Gao and Ganguli, 2015; Mastrogiuseppe and Ostojic, 2018).

<u>Concerns about mean response vector analysis</u>
**The authors' main findings are largely dependent on analyses of the mean response vector. This vector is calculated by averaging the activity of neurons across 600ms-1s of imaging data. Authors don't provide reasoning for this choice in window size. I'm curious how variable the mean response vector is over the course of this time. Authors should quantify this movement by using a sliding window and comparing the movement over time within a 1 second interval to across session movement. I'm especially concerned about the robustness of this vector. If you hold out a subset of trials (odd/even or first half/last half) and calculate the mean response vector within one session, how much does it change? How does that compare across sessions? If this change doesn't accumulate across sessions, I wouldn't refer to this phenomenon as 'representational drift'.

Does the rate of change of the mean response vector change over the course of the imaging period? I would like to see a quantification of 2c for all mice. For example, it appears as though repeats 4 and 5 are more dissimilar than 1 and 2.

Line 158, 395 The authors often report that mean is correlated with variance explained, but doesn't this have to be true for neural activity (which doesn't go below zero)?

Include experimental data for comparison in 5d-i, 6.

Minor Concerns:

Line 30: Also provide examples of stability in connectivity (Holtmaat and Svoboda, Nat Review Neuro 2009)

Line 38: Not all refs in 3-12 show representational drift. Remove [4,6,7] from this citation.

Line 38 Include recent work on bats as a counter example (Liberti 2022)

Line 55 : Define 'magnitude of neuronal representations'

Line 90: Drift is across days to weeks, not seconds. Is there a citation for ‘seconds?’

Lin 99: Include citation to [Michael E Rule et al (2020) Stable task information from an unstable neural population.]

Line 43: Central pattern generator doesn’t encode a representation. It generates muscle patterns. This is a confusing reference to [15]. This system is robust to differences in neural activity. Maybe just change the wording here.

figure 1 legend: ‘PCA decomposition’ is non standard language. Change to ‘we perform PCA’

Line 293: Unclear what a ‘group’ is

298 previously

343 What are network dynamics in a feedforward network?

474 Sparsity is different than low dimensionality

486 What is the reasoning for sparsity to target active neurons rather than less active?

540 Citations or examples for more realistic network models?

582 tells

604 Natural

663 typo? I couldn’t parse.
