# Supplementary material for: The geometry of representational drift in natural and artificial neural networks
Source: PLoS Comput Biol. 2022 Nov 28;18(11):e1010716. doi: 10.1371/journal.pcbi.1010716 (PMC9731438; doi:10.1371/journal.pcbi.1010716)
Supplement: Attachment — Submitted filename: Representation_Drift_review_response.pdf [file pcbi.1010716.s012.pdf]

Reviewer Response: The Geometry of Representational Drift in Natural and Artificial Neural Networks

Kyle Aitken[1], Marina Garrett[1], Shawn Olsen[1], and Stefan Mihalas[1]

[1]Allen Institute
MindScope Program
Seattle, Washington

August 29, 2022

We want to thank all reviewers for the thorough feedback. With the feedback that has been implemented, we believe the clarity and overall message of our paper has been greatly improved. Below we have discussed our attempts to address all concerns of each reviewer. Reviewer feedback is in *purple text* and our responses are in black text. For longer responses, we have bolded descriptions of the changes that we have made to the paper. Note some changes were made to the manuscript at the request of PLOS (e.g. adding author summary and splitting off the supplement).

Reviewer #1

Authors define geometry of neuronal representations to better under stand how they drift. Authors find that neural representations change the most in dimensions with highest variance. This geometry of drift in the visual cortex appears to be similar to drift in ANNs with dropout. The authors conclude with a hypothesis that drift could help prevent overfitting. It is interesting that the rate of drift was similar across familiar and novel stimulus presentations.

Representational drift is a feature of neural data that was recently reported in the hippocampus and cerebral cortex. This feature must be quantified and characterized in order to better develop an understanding for potential roles in neural computation. Therefore, the goals of this work are interesting and important. However, authors' methods of characterization are limited in providing further insight beyond what has already been reported. Importantly, there's little evidence for representational drift happening at all in this work. More careful comparison across sessions 1 and 2 compared to sessions 1 and 3 will be crucial to verify that representational drift is measurable in this data. Additionally, the contribution of changes in behavior across sessions was not addressed. While previous work has shown that behavioral confounds don't explain all neural change associated with drift, it is important to examine the contribution of behavioral change in any careful analysis of neural data. The dataset analyzed in this work contains behavioral information that could be analyzed for a revised draft. Extensive work must be done to demonstrate accumulation of change over time associated with drift, and also robustness controls for the mean response vector. These revisions are possible given the previously collected data; therefore, major revisions should be required before accepting this work for publication.

Major Concerns:

**** Based on previous work, we might expect the magnitude of drift to increase as the gap between sessions increases. I couldn't find evidence of this anywhere in the paper beyond an example figure in 2c and d. In line 168, authors state that an SVC can distinguish separate sessions. Does decoder performance improve with a larger session gap? (1-3 should have higher performance than 1-2 or 2-3)? This does not appear to be the case (or is very weakly present) in Figure 2e across different stimulus groups. How does the same plot look*

for the same stimulus groups (summary figures for 2c and d)? How does the rate of change in 2e compare to previous studies (ie. Xia J, Marks TD, Goard MJ, et al. (2021) etc)? More direct comparisons to previous work would be helpful.

Indeed, we were also surprised that we have found that the amount of drift seems to change very slowly, for the time scales we are observing (though we do not believe this to be true for shorter time scales). This certainly does not quantitatively match findings in other works, but, as we discuss in more detail below, there does not seem to be a consensus about the speed with which the drift of excitatory cells in the visual cortex continues to "accumulate" over time. **Given the difference with some other findings, we have tried to more precisely qualify our statements when it comes to these findings throughout the work.** In line with our other findings, we do not find the decoder performance significantly increases with the larger session gaps (1-2: 0.68 +/- 0.09, 2-3: 0.68 +/- 0.08, 1-3: 0.69 +/- 0.09). **These results have been added to the text.**

Note when we say "across stimulus groups" for 2e, we mean that we average the change in the response vector for each individual stimulus group over all stimulus groups. That is, we are not comparing the response vectors of different stimulus groups in this plot. At no point in this work, other than figures like 2c and 2d (the primary purpose of which are for comparison to Deitch et. al.), are we comparing the representations of different stimuli. **We have changed the language in Fig. 2's caption to make this clearer.**

We have made an attempt to provide comparisons to other works below, especially those that also study drift of excitatory cells in V1. **We have added a summary of this discussion to the Discussion section.** Almost all works use slightly different measures to determine similarity of representations. We also note the rate of exposure to other stimuli is different in our work than other works. For example, in the Passive data we often have 3 exposures in ≤ 9 days, with significant exposure to other stimuli during imaging sessions, the order of which change from session to session (Fig. 1b). For the olfactory cortex, it has been shown that more frequent exposures to stimuli stabilize the representation (Schoover et. al), so the difference in stimuli exposure may be another reason our results differ from other works. Although there are different measures used for similarity in these works, there does not seem to be a consensus that drift continues to "accumulate" over time.

- Xia et. al. study drift in excitatory cells of V1, with a less frequent exposure to stimuli ($\sim$1/week) than what we study. For their similarity measure, they use the correlation coefficient and average the dF/F response over the entire 30s trial. They observe a decrease in mean similarity over several weeks, but overall it is fairly small and seems to actually stabilize after a certain time (Fig. 1c). Error bars do not seem to exclude a correlation that remains constant over time. Note that we only include data from 1-9 days, whereas they include data up to 5 weeks of separation. We also note that our initial correlation of representations is generally smaller than their shortest time scale (see Fig. S1 of our paper), with ours on order of what they find for their longest time scale.

- Dietch et. al. analyze the same dataset as our work, what we refer to as the Passive data, but notably continue to fit for long time scale separations (>9 days) that we omitted due to small sample sizes (note this amounts to an omission of <5% of the data). They also use a slightly different measure of representation than we consider: they either create a single response vector for the entire movie repeat, or construct a tuning curve to represent a neuron's activity over a repeat. For V1, they see a relatively small decrease in correlation for each of these measures (Figs. 3J and 3K). Notably, the majority of the data points for 10+ days, which we omitted, exist below the linear fit.

- Marks et. al. also investigate drift in excitatory neurons of V1. Again, longer time scales and less frequent exposures to the stimulus are used in this work ($\sim$1/week). They also use a correlation-based measure in this work based on averages of neuronal activity over the entire trial that is also calculated relative to the within-session correlation. For the natural movie data, their drift metric shows dissimilarity slowly increases for the 2-5 week marks, after which it becomes relatively stable once again (Fig. 1i).

- We can make comparisons to works that investigate other parts of the brain, but these of course should be taken with a grain of salt. For example, Driscoll et. al., which focus on the parietal cortex and also use different measures for similarity. However, the fraction of cells with consistent peak location (Fig. 1B) does appear to somewhat stabilize for time scales between 10 and $\sim$25 days (as they note, large

intervals have fewer mice, so it is hard to tell if the decrease for very large intervals is indeed due to further drift or because the sample group has changed).

***The authors seem to have missed an important line of work that is highly relevant to their findings from Denise Cai's group. This work shows that there is an 'excitable pool' of cells that varies over days. The neurons that are most active are part of the excitable pool on any given day. As the excitable pool changes across days, neurons that were most active, are no longer active on subsequent days. These findings would result in a phenomenon similar to dropout. Authors should reference this work extensively throughout (Cai 2016, Mau 2020, etc).*

Thanks for bringing this work to our attention. We agree, it's highly relevant to the dropout-like behavior we are proposing and as such **we have added a short discussion of this work and its relevance to the Discussion section.**

*** Importantly, behavioral measures were not included at all in this entire work. The available datasets include metrics like running speed, eye position and pupil area. These variables should be analyzed as extensively as the neural data and included in all session comparisons. For example, in all decoding analyses, is it possible to decode which session a dataset came from based on running speed alone?*

** Line 170 : Authors cite representational drift work in hippocampus [11] as not being linked to behavioral features and claim that this is reasoning to not analyze such data in visual cortex. This explanation is not sufficient, especially given several works citing the relevance of behavioral features when analyzing neural data (Musall et al 2019, Clopath et al 2017, Liberti 2022).*

We have added an additional analysis of the behavior of the animals we study to the paper. See the new Sec. 4.3.1, in the supplement of the paper for details. Specifically, we analyzed the Behavioral data as this is where many of our measures were most robust and matched the rest of the results found in the paper. The influence of behavior on the Passive data has been analyzed previously in Dietch et. al., where they found behavioral metrics (running speed, pupil area) could not explain the drift they observed. As suggested, we added a decoding analysis to see if it is possible to decode what session is being observed from the behavioral metrics of said session. Notably, the trained classifiers perform at chance levels in both the familiar and novel data, which we take to mean there is no significant difference in behavior between sessions that might explain the characteristics of drift we observe.

Additionally, we have analyzed if the change in behavior from one session to another can explain the amount of drift we see for each mouse. Fitting to the change in all three behavioral metrics discussed above, we do not find significant evidence that the amount of drift can be explained by change in behavior across mice. (See text for additional details.)

All comparisons between odd and even trials should also include a comparison to the first and second half of sessions in order to determine whether there was any drift recorded over a single session (ex. in line 169 and 282). Additionally 3e,f should have odd even/ first half second have same day comparisons.

We have added comparisons between the first and second half of sessions in the places requested. The SVC classifiers between first/second half of sessions, on average, achieve slightly higher accuracy on first/second versus even/odd, but both are significantly lower than between session classification and still quite close to chance levels.

Note the even/odd and now first/second null examples are used to compare against the SVC classifier that distinguishes the groups across sessions. These quantities are computed for each individual group (e.g. 1s blocks or hit/miss trials) and averaged together. What is shown in 3e and 3f are SVCs used to distinguish the different stimulus groups within the same session, and how they might differ from session to session (so different groups have different labels).

The null data we have tried to use in these plots are that of the randomly rotated drift (the '–' lines in Figs. 3f and 4h). We take the fact that the accuracy doesn't change significantly with a random drift direction to mean the persistence is more a result of the high dimensionality of neuronal space and not the particular direction drift is occurring. The even/odd or first/second half null examples don't seem to help us convey this message. We could investigate how a classifier used to distinguish groups in the first half of a session might differ from the second half of a session, but because it would only be training on half the data compared to across-sessions, this also may not yield a strong comparison (especially in the case of the Passive

data, where we would be working with test+train set of total size 10 for each mouse). If this is something that you believe is truly needed for a proper comparison, we could add it but we believe such a comparison is not needed in this case.

Line 9: The idea that neurons represent external stimuli is a hypothesis and should be stated as such. (Brette, 2018; Freeman and Skarda, 1990) The authors talk a lot about representations but make no effort to link neural activity to any particular representation. Given (Musall et al 2019) it will be important to determine which external stimuli and behavioral variables are contributing to neuronal activity.

We have added the relevant references and qualifier in the Introduction. Note that we do train SVCs to distinguish representations from the neural data in both experimental setups and we do find they are able to achieve greater than chance percentages. Regarding how behavioral activity contributes, see discussion of behavior analysis above.

Line 248: Authors state that decoding accuracies are higher than in previous work [8,9]. However, my understanding is that the timescale for this work is much shorter and it is particularly difficult to compare results to [9], where authors performed an 8-way classification. What is the actual accuracy? The results in the present study are obfuscated by plotting relative accuracy. A more direct comparison to previous work would be more informative.

You're correct, comparison to [9] is not quite a setting where we can directly compare to results from other works, especially since the task in this work is much simpler. **We have changed the wording of our comparison and also added a longer discussion of the details of these results in the main text (see below for details). We have replaced the relative cross accuracy plots (3f, 4h, 6b) with the just cross accuracy plots for the Passive, Behavioral, and CNN data.** Here is a direct comparison to previous work.

- Schoonover et. al. observe a significant decrease in cross-session classification accuracy, even for their shortest time scale of 8 days (Fig. 2c). Notably, their task is significantly more difficult than the one we consider here, where they do 8-way classification and only test on 41 units. They also observe relatively stable same-day classifier accuracy over time, for the time scales we consider in this work. Both of these work against the hypothesis that cross-classification accuracy remains high as a result of the high-dimensionality of neuronal space.
- Driscoll et. al. also investigates population decoding accuracy for a 2-way task, left or right movement (though note earlier differences in setups). They find decoding accuracies remain stable across days when either (1) the cell count is high (100) or (2) they decode on a small number of neurons that are the most consistent activity-behavior relationship (though notably, this is both dependent on the mouse's location in the T maze, and seems to deteriorate for time scales greater than what we investigate).

Authors claim that the number of days between sessions is only weakly correlated with drift starting at line 179. I don't see any comparison between the number of days between sessions compared to the number of sessions, as was reported in [9]. The authors should do a more thorough analysis as in figure 4d of ref [9] before making these claims.

Although the effect of presented stimuli on representational drift as presented in [9] is highly interesting and relevant, the number of stimuli sessions between trials for both the Passive and Behavioral datasets we consider in this work are much more uniform. Neither of these experimental datasets have a number of stimuli exposures (daily exposure over 32 days) or significant irregularity (familiar vs. unfamiliar, the latter which was presented 8 times less frequently) that was studied in [9].

- For the Passive data, except for failed trials (of which there are very few) sessions 1, 2, and 3 are not separated by any additional presentations of the relevant stimuli. At most, we'd be able to investigate the effect of the additional imaging session between 1 & 3 compared to 1 & 2 and 2 & 3, but as mentioned above we did not see any quantitative difference in the drift between these cases when we trained an SVC to distinguish them.

- For the Behavioral data, the two imaging sessions are usually separated by a single "passive" session where the mouse sees the stimuli but is automatically given rewards. Any mice that had significantly irregular testing schedules were omitted from the analysis.

Another reason why comparison to [9] is difficult, is that the stimuli we analyze are very similar to natural stimuli. As mice continue to use their visual system even if there are no recording sessions, the images represented by the visual cortex in between the recordings will be wide not not under experimenter's control.

When reporting low dimensional subspaces, authors should cite other work that has reported similar properties (Gao and Ganguli, 2015; Mastrogiuseppe and Ostojic, 2018).

Added these citations after mentions of small D/n for experimental data.

Concerns about mean response vector analysis

***The authors' main findings are largely dependent on analyses of the mean response vector. This vector is calculated by averaging the activity of neurons across 600ms-1s of imaging data. Authors don't provide reasoning for this choice in window size. I'm curious how variable the mean response vector is over the course of this time. Authors should quantify this movement by using a sliding window and comparing the movement over time within a 1 second interval to across session movement. I'm especially concerned about the robustness of this vector. If you hold out a subset of trials (odd/even or first half/last half) and calculate the mean response vector within one session, how much does it change? How does that compare across sessions? If this change doesn't accumulate across sessions, I wouldn't refer to this phenomenon as 'representational drift'.*

We agree that our reasoning for using these particular windows was not well motivated in the text, and have added explanations for our choices. The 1s window of averaging for the Passive data is for direct comparison/confirmation of the results of Dietch et. al., who analyze the same data and use a similar window. The 600ms window for the Behavioral data is meant to match the experimentally defined "response window", the time window where we look for the mouse to lick in response to an image change in order to solve the task, which is chosen to match the time-scale of the image change task.

Note the response vector (RV) is indeed variable over the time windows we investigate, but for both datasets we do not believe comparing this variability to the between-session drift magnitude yields useful measures:

- For the Behavioral data, since the stimulus that is invoking the responses changes with time, using a rolling window to calculate the RV would certainly yield variability. However, part of this variability is most certainly due to the mouse's response to the change in stimulus image, which is distinct from variability we are trying to capture in this work for the same stimulus due to the passage of time. We do our best to eliminate variability due to stimulus and so all computations of drift in this work are done for RVs corresponding to the same type of stimulus. Thus we do not believe the variability within this window should be compared to the variability due to drift, since these would likely be capturing distinct sources of change in neuronal responses.

- For the Passive data, computing the variability of the RVs using a sliding window would capture a significant amount of noise in each of the individual response vectors, which we again believe would yield results that are difficult to compare to the between-session drift we are attempting to analyze in this work. This is a large reason for us considering long time scale drift: our ability to capture many responses to the same stimulus within a relatively short amount of time gives more robustness to the mean responses vectors (see below), and thus drift. This is notable in the distribution of RV angles and correlation coefficients for, say, short time scale drift (30 to 270 seconds, computed with individual RVs, red) versus long time scale drift (1 to 9 days, computed with mean RVs, blue). The short time scale drift does not benefit from the averaging of response vectors over many measurements and clearly yields a very different distribution:

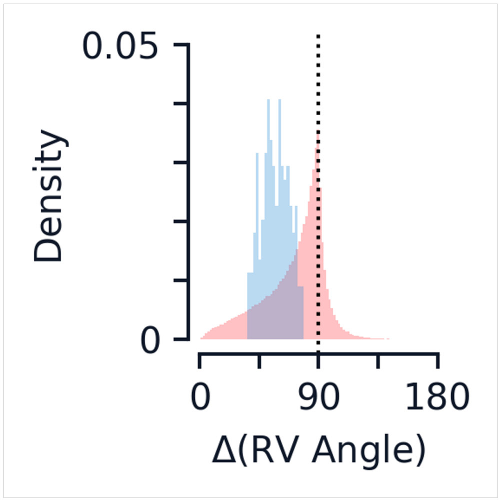

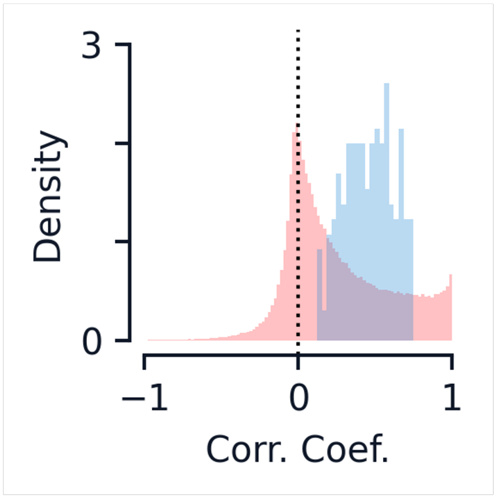

As suggested, we investigated the robustness of the response vector (RV) while holding out a subset of the trials. **We have added a section to the supplement (4.3.3) discussing these results.** For the hit trials, we find the change in the RV to be fairly small: leaving out half of the data that contributes to the mean RV changes the vector by about 20% (as measured by magnitude of change over magnitude of mean RV). The magnitude of drift is comparable to the magnitude of the RV, so the size of the change in RV has the same relative size (∼20%) to the change in the mean response vector across sessions. The robustness is less strong for groups with smaller sample sizes (e.g. miss trials ∼40%) or the Passive data which only has 10 members for each group. Since we found the drift to be similar in size to the response vector (Fig. 2f, S2e, S2i), the intra-session variability is approximately 5 times smaller than the between session drift for the hit trials. In all cases, the robustness of the drift results are strengthened by the number of mice we look over. Additionally, in the Passive case, the robustness of the drift results come from the large number of stimulus groups from which we can measure changes in the response vector. Notably, these less robust cases agree with the most robust results (the Hit trials). Additionally, in the supplement, we analyze drift for change/no change data, which benefit from an even larger number of trials, and continue to find similar results. Altogether, we believe this backs up the robustness of the results we have found in this work.

Does the rate of change of the mean response vector change over the course of the imaging period? I would like to see a quantification of 2c for all mice. For example, it appears as though repeats 4 and 5 are more dissimilar than 1 and 2.

Below is the plot you requested (both zoomed in to the first 5 repeats, and all 10 repeats). Visually, the increased similarity between 1 and 2 versus 4 and 5 seems to have vanished. Let us know if you would like us to further quantify how the rate of change of the mean response vector changes over the session.

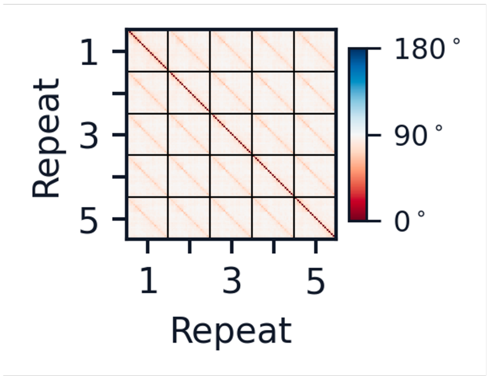

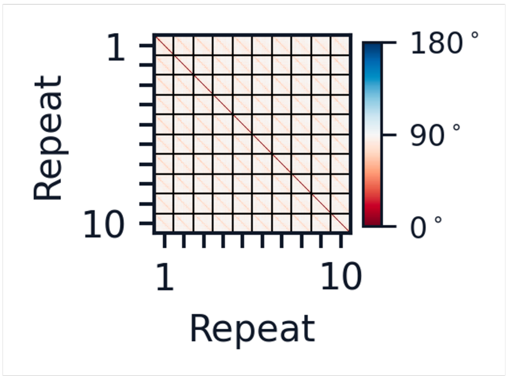

Line 158, 395 The authors often report that mean is correlated with variance explained, but doesn't this have

to be true for neural activity (which doesn't go below zero)?

This is certainly known to be a property of neural recordings and Poisson-like processes in general. It is not intended to be presented as something novel to this work, the goal here is to confirm this finding and point it out since it is useful to understand the relation between means and variance. Given that the drift vector is a difference in means of RVs with this property, the change in means also has implications on the variational space of the group.

Include experimental data for comparison in 5d-i, 6.

We added experimental data from 4c-e to 5d-i and to 6 where relevant. 6c,d have no experimental equivalents. For 6e, the purpose is for comparison of targeted node dropout to uniform node dropout, so we felt adding experimental data to this plot cluttered it too much (and we have already confirmed in the previous page that experimental data matches node dropout quite well). For 6f, the purpose is to show the change in RV angle stabilizes after a certain time, and so showing a comparison to the change in RV angle for experiments isn't relevant for what we are trying to demonstrate (and also, the equivalence of dropout rates between days/epochs is unclear).

Minor Concerns:

- *Line 30: Also provide examples of stability in connectivity (Holtmaat and Svoboda, Nat Review Neuro 2009)* Done.
- *Line 38: Not all refs in 3-12 show representational drift. Remove [4,6,7] from this citation.* Done.
- *Line 38 Include recent work on bats as a counter example (Liberti 2022).* Done.
- *Line 55 : Define 'magnitude of neuronal representations'* Now 'magnitude of representation vector'.
- *Line 90: Drift is across days to weeks, not seconds. Is there a citation for 'seconds?'* Deitch et. al. 2021 study drift between movie repeats that are 30-40 seconds long, however 'minutes' is probably a more accurate characterization of the timescale, so this has been changed.
- *Lin 99: Include citation to [Michael E Rule et al (2020) Stable task information from an unstable neural population.]* Done.
- *Line 43: Central pattern generator doesn't encode a representation. It generates muscle patterns. This is a confusing reference to [15]. This system is robust to differences in neural activity. Maybe just change the wording here.* Separated into two sentences and changed wording to make this clearer.
- *figure 1 legend: 'PCA decomposition' is non standard language. Change to 'we perform PCA'* Done.
- *Line 293: Unclear what a 'group' is* Changed to 'stimulus group'.
- *298 previously* Done.
- *343 What are network dynamics in a feedforward network?* Changed to 'network's training'.
- *474 Sparsity is different than low dimensionality* Changed to 'dimensionality'.
- *486 What is the reasoning for sparsity to target active neurons rather than less active?* Again, we think we are overusing "sparsity" here, what we meant was this may be an attempt to minimize metabolic cost of the representation and changed text to reflect this.
- *540 Citations or examples for more realistic network models?* Added example (spiking neural networks) and citation.
- *582 tells* Fixed.
- *604 Natural* Fixed.
- *663 typo? I couldn't parse.* Reworded.

Reviewer #2

================Summary================

The paper studies representation drift using neural recordings and artificial network dynamics. The former shows the type of geometrical changes in neural activity, the latter tests different types of noise in order to reproduce those geometrical changes. The authors find that node dropout is the most likely candidate. The paper is well-written and, to my knowledge, the results are novel. Moreover, the suggested link between representation drift can facilitate both experiments (as the results suggest a very specific type of noise) and theoretical studies of how noise affects neural representations.

================Questions================

Neural data question: as you have access to neural recordings, wouldn't it be possible to check for node dropout? You've tested relatively high dropout rates (>10%), which should be detectable from data.

We certainly have the raw fluorescence recordings of the neurons, but because fluorescence only spikes when a neuron fires, it would be difficult to disentangle a neuron truly "dropping out" due to some sort of random masking from a neuron's response simply being quiet at the given time step because the stimulus response/other brain areas do not excite it. It would be very interesting to further investigate neuronal recordings/connectome data to see if there is credible evidence of randomized dropout but we believe this is outside the scope of this work since it would take quite a bit of analysis of the noise structure of the neural data and how dropout would differ from other null models of noise. Additionally, our CNN model is a crude approximation for the visual cortex, so although we observe that 50% dropout in our model best matches what we observe in the brain, we are not claiming the brain itself has that high of a dropout rate (notably, from the metric we use in Fig. 5a, even smaller dropout rates are better fits to the experimental data than other types of noise).

I think the deep learning side of the paper can be significantly improved.

First, the networks chosen in experiments are not trained to convergence (as you run SGD with a constant, rather than a decreasing, learning rate). If you train to convergence, your network might become more sensitive to noise during the second phase of learning, and potentially change the results. (Note that this is based on my intuition and may not be true. However, it's always good to know if your results can be affected by tweaks in the learning procedure.)

We agree that more sophisticated learning schedules, especially one that decays learning rate, could lead to a better converged network state. However, our goal in this setting was to not train the network to perfect convergence, but rather train the network until the feature layer activity resembled neural data and the network had reached stable performance (and not necessarily stable weights). This setting was motivated from the fact the brain's neural connections are always in flux and representational drift is known to occur over time, even during stable performance on tasks. We believe the fact that the networks reached stable accuracies (Fig. S4b) that are well above chance was enough for this goal. As we note in the text as well, we ensure the networks are not overfitting the train set by carefully monitoring the test loss. **These goals were not well pointed out in the main text, so we have clarified it in the paper in footnote 8.**

Note training with a decreasing learning schedule would also make investigating the networks in the continual learning setting more difficult. This is because in order to continue to observe the networks at the steady state they ultimately reach (again measured by accuracy/loss not weight convergence), we would need to train at the learning rate at which they converged.

Second, it would be good to know your results are not sensitive to the number of feature neurons. You can try scaling the whole network, or just the feature layer (e.g. 2x, 4x) to see if the changes are consistent across network sizes. Scaling the whole network would also improve CIFAR10 performance (the current 60-70% is rather far from the standard 85-90% you get with a ResNet18), although I don't think the exact accuracy is important for this study.

We further investigated how the CNN results may depend on the feature layer size. We indeed found that for both smaller and larger feature layers (56 and 126, respectively), the qualitative results we found for noise injections of node dropout and additive Gaussian persist (see figures below). Although there are minor quantitative differences, the qualitative results and their distinct drift are the same as what was observed for the original feature layer size. **We have added additional text to the paper discussing these results.**

Increasing the feature layer size did slightly increase the accuracy. As you note though, the goal in this work was not to reach high CIFAR10 performance. Notably, the different groups of the neural data (e.g. the Hit/Miss groups) are by no means perfectly separable (we find they can only be distinguish from neural data at about 70% accuracy). We felt the noisier representations of the CNN architecture that yielded smaller accuracies were actually closer to what was observed in the neural data rather than training to the highest possible accuracy (where the different classes would be easier to distinguish).

Feature layer size 56, dropout: (Note these plots are the same as those in Figs. 5d-5f and 5g-5i, these just haven't been touched up as much. The red and green correspond to two different classes. If needed we can also provide numbers associated with fits.)

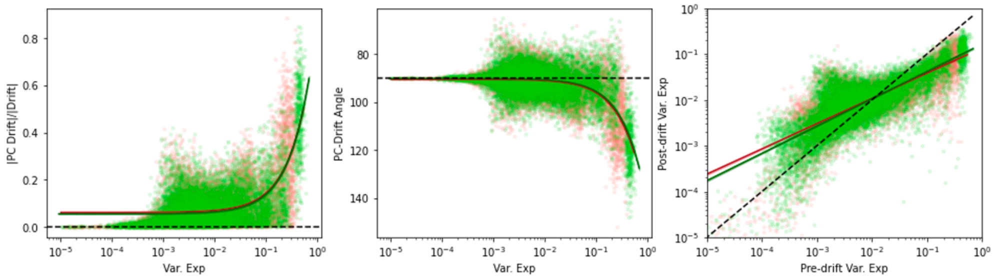

Feature layer size 56, additive gaussian noise:

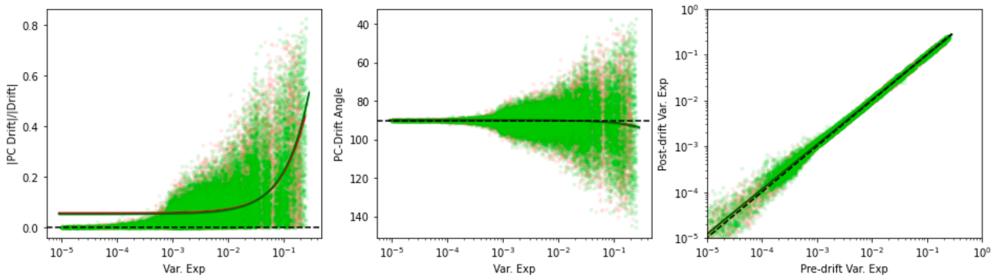

Feature layer size 126, dropout:

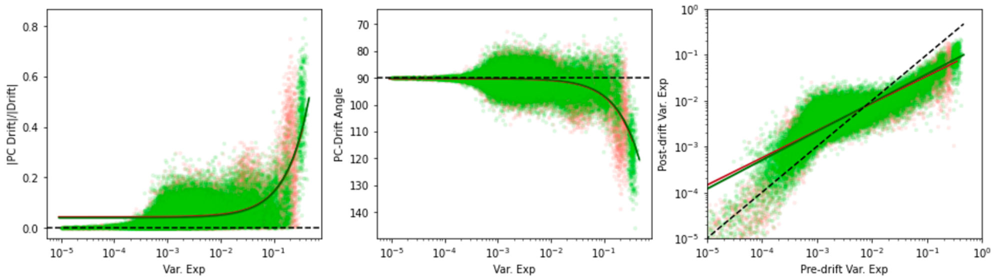

Feature layer size 126, additive gaussian noise:

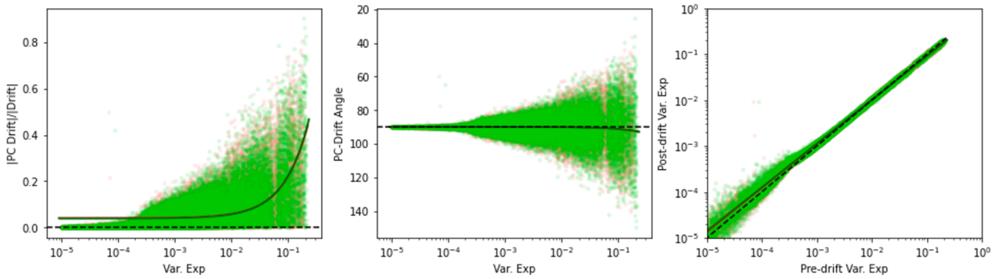

Third, are your results specific to continual learning? You can train the network as you did, and then run the same experiments with re-drawn dropout noise, but without training. This setup would be somewhat closer to the passive data.

The results are specific to continual learning. Our reason for considering this setting is that drift in neural representations is always investigated in a setting where the animals continue to interact with the task and world around them, so we felt that investigating the CNNs in a setting where they continue to be influenced by the training data (but notably maintain persistent accuracy) best matched this setting.

We investigated drift in a setting where training is frozen but dropout noise is redrawn. Since the exact draw of dropout noise indeed affects the feature space representation, we do indeed see a "drift" in the representation (see figure below). Notably however, the drift is distinct from that due to dropout in a continual learning setting, and does not resemble the experimental data nearly as well. This was a great idea to confirm that the continual learning setting is important for the results we observe and as such **we have added additional text to the paper explaining these results.** (Again, red and green are two different classes.)

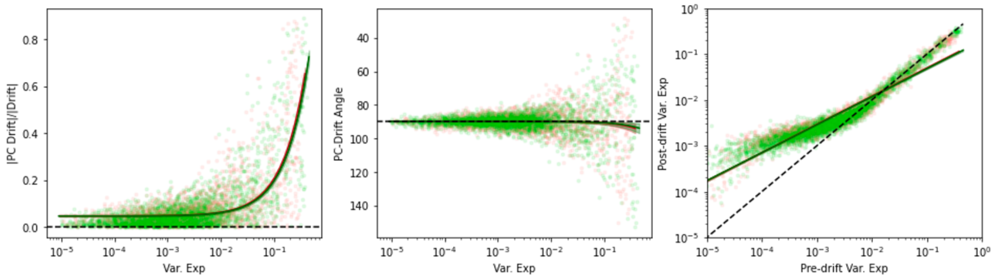

Finally, we note that we already partially control for variation due to re-drawn dropout noise (and noise in general), since in each test set we use to compute drift we average each representation over 10 draws of dropout noise.

================Minor comments================

- *Lines 44-46: citations for both sentences would be helpful.* Added.
- *L164: 5-fold CV on what subsets of data?* We label the response vectors of a given group by what sessions it is from. Data is averaged across all mice and all groups. We have added clarifying text to the supplement about the details of this.
- *L242: citation [8] should be explained rather than just stated.* Added a short qualifier for the citation indicating that this has been pointed out before in said reference.
- *L287: the sentence under (1) reads weirdly, maybe "a drift magnitude of the same order as the mean response vector" would be correct?* Reworded.
- *L360-361: "both" refers to three options in the sentence.* Removed "both".

- *L494: change"s"* Fixed.
- *L508: that's not quite true. ResNet, arguably the most popular architecture, doesn't use dropout. Changing "almost all" to "many" would make the statement more accurate.* Fixed.
- *L509-510: citation needed, even though it's more or less common knowledge.* Added.
- *LFig. 5 (and similar figures): why don't the plots reach 1 on the x axis (variance explained)? I'm assuming it's because your discarded very small eigenvalues (as mentioned in Methods), but Fig. 5 plots stop surprisingly far from variance explained = 1.* Note these plots are of the ratio of variance explained associated with each individual PC, which is bounded between 0 and 1 (inclusive). In general, many PCs are needed to explain the variance of the response vectors, and so all their ratios are <1 by quite a bit. If a single PC explained all of the variance, which was never the case in the neural/CNN data we investigated, then it would have a value equal to 1. We realize that "Var. Exp." is often shown to describe the cumulative variance explained by increasing amounts of PC directions, **so we have modified all x-axes with this label to say "Var. Exp. by PC_i" to try to indicate it is for a single PC direction at a time**.

Reviewer #3

Aitken et al. present a study of representational drift by analysis of experimental recordings in the mouse visual cortex (Allen Brain Observatory data) and simulations of artificial neural network (ANN). The focus of the study is to investigate the geometrical means by which the neural space is transformed in the process of drift. The data is presented in detail and from many different aspects, and some properties of the drift shown here are interesting and thought provoking. Additionally, the finding that the node-dropout method in the ANN can replicate many features of experimental drift is very interesting. However, there are some ambiguities and apparent contradictions in the results that require further explanations (see below).

1- The observation that there is a weak dependence of the drift on the time difference between the sessions is not very clear. In Fig. 2e, the small slope of the plot is understandable but the large intercept (around 60 degrees) makes the result counterintuitive. The plot suggests that, irrespective of the days between the sessions, there is always around 60 degrees angle difference between the mean response vectors for a given group. In other words, it appears as if the drift happens abruptly as a function of time and then stays constant. This is in apparent contradiction with the general notion that drift is a gradual and ongoing process as reported in previous studies. Specifically, in the study of Ref [11] that is based on the same data, a gradual continuous drift was found. Please explain.

This is indeed a point we were confused about initially. Notably, the smallest time scale we investigate is drift over at least one day. Thus, plots like Fig. 2e, do not show how drift would behave on shorter time scales. We indeed expect that, for shorter time scales of drift, the angle will not be 60 degrees (in the limit of zero time difference, it should be zero degrees). We did not investigate shorter time scales in our work, but we did observe this trend in the CNN experiments we performed (Fig. 6f). What appears to be the case from our findings though is that above a certain time scale, certain properties of drift such as the magnitude/change in angle, vary slowly, and may even remain constant. This is indeed somewhat different than other works that have found that, to a degree, drift can "accumulate", though many find drift does eventually stabilize (see response to Reviewer #1 for more detailed comparison, **that we have also added to the Discussion section of the paper**).

We hypothesize that the constancy of certain properties of drift arises from two primary factors: (1) the high dimensionality of neuronal space and (2) the size of neural representations responses being bounded from above and below. A very crude model of what drift may look like under these conditions would be performing a random walk on a high-dimensional sphere, where the position on the sphere is the representation at that given time step. (We choose a sphere so that the representations have bounded magnitude.).

Below are results from such a simulation for a random walk on a 100-dimensional sphere (quite a bit smaller than most neuronal spaces we investigate). The colored lines are 50 trials of the walk, while the solid black line is the average distance/angle from the starting point/representation. After a certain number

of steps the drift of the representation (change in current position minus start position) has, on average, constant magnitude (i.e. drift magnitude) and a constant change in direction (i.e. drift angle). Going to higher dimensions yields even less variance around the mean values.

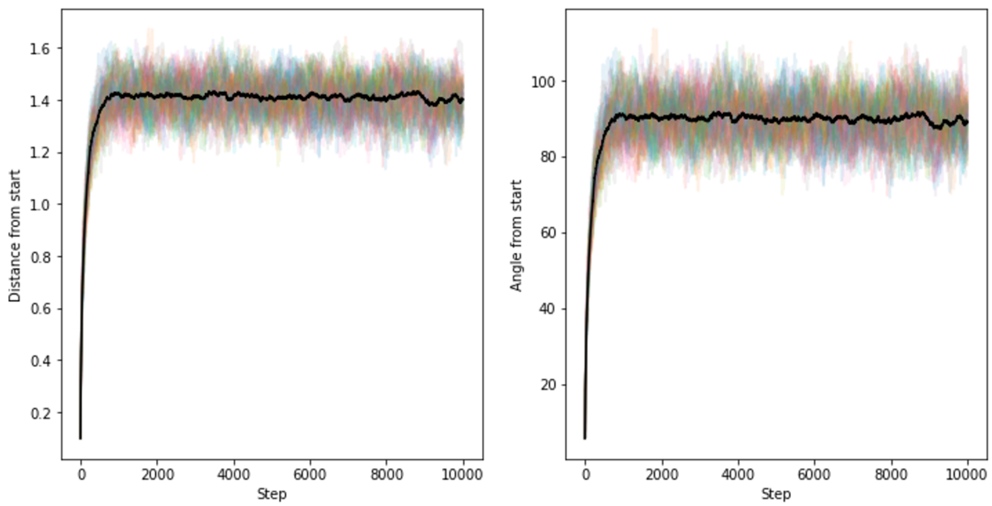

2- The findings that there is a decrease of later variance explained along the top PC directions (of the previous session) and an increase in later variance explained for lower PC directions are interesting. However, a more complete geometrical intuition could be provided here. The authors explain that the variance flows out of certain dimensions to others. Is that also happening with respect to the PC directions of the current session? If that's the case, the D metric (variational space dimension) should decrease from an earlier to a later session which does not seem to be the case from Fig 2g. Please clarify. As a possibility, a rotation of the data from one session to another could explain the increase and the decrease trends noted above while D stays fixed. Please explain if that could be compatible with the data.

This is correct, indeed there is a flow of variance between PC directions with respect to the late session as well. In fact, simply flipping the x and y axes of figures like 3c and 4e can show you this occurs. If this is done, the fits are now above the diagonal for high variance explained and below the diagonal for small variance explained, showing the flow of variance into certain directions for the later session. Note that it is slightly circular to say that the top PC directions of the later session gain variance, since the only reason they are the "top" PC directions of the later session is because they have received a large amount of variance under drift. In other words, they were not the top PC directions prior to drift occurring, so drift does not lead to directions with a lot of variation gaining more.

If drift instead caused variation to flow into the top PC directions of the earlier session (and out of lower PC directions), this would lead to a sparsification of the representation and hence a decrease in D.

It is hard to say if a rotation of the data alone could be compatible with the findings. We have observed that drift has a tendency to flow in a direction obtuse to directions with large variation, and this would not be the case for all possible rotations. We would love to develop better theoretical models and explanations of how drift occurs in neuronal space (the random walk on the sphere discussed above is one of them), but we believe such findings are outside the scope of this work.

3- In this study, the variational space plays a central role in characterization of the drift. A natural question is to what extent the defined variational space represents the drift itself in the short term. Given that Ref [11] also found that drift occurs within minutes in the same data, it could be a possibility that at least part of the variation we observe within different repeats of a given block in a session is due to the drift. In that case, it is not surprising that the between session drift is primarily along the top PC directions. Did the authors see any trend within the variational space (10 data points) that suggests there is a within-session drift? If that's not the case, how do they explain the observed relationship between the drift magnitude and the variation explained along PC directions?

Our metrics here are certainly susceptible to capturing within-session drift and thus, to a degree, the variational spaces of groups contain this variation. In addressing the concerns of Reviewer #1, we compared the features of the short timescale drift within a single session to the between-session drift we study in this work. To study short timescale drift, we treated the first and second half of the engaged trials of a session separately. When we investigated how well an SVC could separate the response vectors from the first and second half of a session, we found its accuracy to be quite close to chance, and thus significantly lower than the SVCs trained to distinguish data from different sessions. This tells us that the separation of response vectors induced from within-session drift is much smaller than between-session drift. **These results were added to the main text to distinguish short and long time scale drift.**

Note that, the direction of the within-session drift does not necessarily align with the between-session drift, and indeed our findings seem to say drift does not always occur in the same direction, since drift does not accumulate over time (Figs. 2e,f). Thus, even if part of the variational space PC captures within session drift, we do not believe this would necessarily imply that between-session drift must be aligned with the top PC directions. We hypothesize that the observed relationship between the variance explained and drift magnitude simply comes from the fact that directions that have the most variance tend to have the largest mean values along said directions (Figs. 1e, S1a, S2a, S4d), and thus have the most activity that can be changed from drifting. Notably, we observed this trend across all types of noise in the CNN (Fig. S5), and so we believe this feature isn't exclusive to the drift features we observe in the brain.

4- For the ANN, please specify when exactly the noise was injected in the process of training. (i.e., for each noise type, did you first train the network without noise until steady accuracy was achieved, and then started injecting the noise from epochs 0-10?) This is not very clear upon reading the text and could be added, for example, to the paragraph ending in line 346.

All networks are trained with their specified noise so that once they reach a steady accuracy and we start tracking the change in representations, there is not an abrupt change in their behavior. **We agree this was not clearly stated in the main text and have added clarifying text immediately below the introduction of the five types of noise.**

5- In the paragraph starting in line 372, please clarify how the variational space was measured/defined for the ANN. I assume that this was measured for the 1000 samples of each class. Was this measured at epoch 0, after a steady accuracy was achieved with training that included noise?

This is exactly correct, we measured each class's variation space over a test set of 1000 samples. This was measured starting at the epoch where we observed the network had achieved a steady test accuracy, and the specified noise was injected during training and during the 10 additional epochs where training continued and drift was measured.

Minor comments:

- *6- Referring to line 157, what is the actual (average) value of D? Since we know it is always less than 10, it would be meaningful to report its actual value as well.* $D = 2.7 \pm 1.1$. Added to footnote.
- *7- Please clarify if the pairwise angle plots in Fig. 2c are from one session only. Also, it appears from the plots that certain blocks (almost the first half) are more stable across repeats, while the other half are relatively unstable (i.e., part of the diagonal line in the plots between the consecutive repeats disappear). Please explain what aspect of data or experimental design could cause such effect.* The plots in Fig. 2c are from a single session, we have added language to clarify this. We note that this is from a single mouse, see Dietch et. al. for more robust results across all mice. It is hard to say whether this is a result of the stimulus (Natural Movie One, which has a relatively smooth opening sequence of images) or a result of this being the first part of the movie the mouse sees.
- *8- In the last paragraph of page 18 (two lines after line 580), by "projection vector" the authors perhaps meant "projection matrix" or "projection operator".* Fixed to "matrix".
- *The experimental data used in the study is publicly available. I am not aware if the computational codes are available.* We added the address of the repo that contains all supporting code, thanks for pointing

this out.
