# Supplementary material for: The geometry of representational drift in natural and artificial neural networks
Source: PLoS Comput Biol. 2022 Nov 28;18(11):e1010716. doi: 10.1371/journal.pcbi.1010716 (PMC9731438; doi:10.1371/journal.pcbi.1010716)
Supplement: Attachment — Submitted filename: Representation_Drift_review_response_2.pdf [file pcbi.1010716.s013.pdf]

Reviewer Response 2: The Geometry of Representational Drift in Natural and Artificial Neural Networks

Kyle Aitken[1], Marina Garrett[1], Shawn Olsen[1], and Stefan Mihalas[1]

[1]Allen Institute
MindScope Program
Seattle, Washington

October 21, 2022

We again want to thank the reviewers for the thorough feedback. Below we have discussed our attempts to address the remaining concerns of the reviewers. We have again bolded descriptions of the changes that we have made to the paper.

Reviewer #1

I would like to thank the authors for their extensive work addressing my concerns and the concerns of the other authors. However, two major concerns remain, preventing me from recommending this work for publication in its current form. The authors claim that, "there does not seem to be a consensus that drift continues to

"accumulate" over time." This statement is simply not true. Drift is defined as gradual changes in population responses that accumulate over time (Rule et al. 2019; Deitch et al. 2021; Schoonover et al. 2021; Driscoll et al. 2022). Instead, authors find a similar amount of change between sessions 1-2 and 1-3. Without any evidence of gradual accumulation of change in this work, I don't think it's appropriate to use the term 'representational drift' throughout. Based on comparisons to Deitch et al. 2021, it seems as though the time frame of this work is not long enough to see changes associated with drift.

We are making no claim in our paper that representational drift does not accumulate over time, just that certain metrics of change used on the data we study in this paper do not change significantly over the time scales. Certainly we acknowledge that shorter time scale drift must accumulate over time and if the responses were taken over longer time scales, perhaps stronger evidence of accumulation would be observed. **We have added footnote 4, where we first introduce these results, to clarify this, and to point toward the larger discussion around this later in the paper**. However, we do not think this invalidates our use of the term "representational drift" since we believe first and foremost, to use the definition of Rule et. al. 2019, drift is defined as "...ongoing changes in these representations that occur without obvious changes in behavior." The fact that certain metrics we observe do not change significantly over time periods of days does not mean the representations stop changing. As we pointed out in our initial response, we are not the only work to observe a stabilization of parameters over time periods lasting several days.

We should also note that this finding was never intended to be a major finding of our paper in order to contradict what is found in other works. It was merely a confirmation that certain drift quantities vary slow enough that we could average metrics across distinct time differences. We realize that having this finding listed in the contributions at the start of the paper may be misleading so **we have removed this finding from the primary list of contributions**.

Several studies investigate representational drift that occurs over comparable time scales to what we study, so we also do not believe that is a shortcoming of our work. Deitch et al. 2021 and Sadeh and Clopath 2022 study representational drift on significantly shorter time scales than we do. Once again, the longer time scale

data that was included in Deitch et al. 2021 was classified as outlier data for us due to the small number of session counts with longer time differences (and these sessions represent ¡5% of the data).

I suggested the authors should do a more thorough analysis of behavioral contributions to the geometrical changes that they report. I was not satisfied with the response, especially given recent work showing behavioral contributions to drift(Sadeh and Clopath 2022). I'm confused and surprised that the authors claim that behavior has little to no contribution on representational drift, especially given the recent analysis of the same dataset. Maybe this has some relation to subsampling the data such that only the most similar trials are analyzed? More direct comparisons to analyses in (Sadeh and Clopath 2022) will be critical.

We agree the findings from Sadeh and Clopath are extremely interesting and that behavioral effects must be taken into account when analyzing drift. **We have added it to the related works section**. Let us point out two important differences with their paper and ours:

1. The entirety of Sadeh and Clopath paper is concerned with shorter timescale drift (hours) and analyzes behavioral effects of drift within the same session. Animals can get tired and/or stressed on the rig, and their behavior can change from the first to the second half of the session. Our paper is concerned with drift on longer time scales (days) and purposely analyzes data that is averaged over entire sessions in an attempt to remove any latent effects such as behavioral dependence. **We have added this important difference in Section 4.3.1 where we discuss behavioral controls**. Importantly, we did not find significant differences in behavior between sessions as described in Section 4.3.

2. The Sadeh and Clopath analysis was conducted on neuropixels data while all of the data we analyze is gathered via calcium imaging. The reviewer's claim that we analyzed the same dataset as them is imprecise. It can be confusing as both datasets are part of the Allen Brain Observatory, however there is certainly a difference in techniques.

We believe a complete characterization of the behavioral effects of drift at the level of Sadeh and Clopath, because such a study has not yet been conducted on drift at time scales of days or longer, is outside the scope of this work. Indeed we believe the controls that we have used to ensure a smaller variance in mouse behavior (only engaged trials are analyzed) is a strength of our analysis.

Reviewer #3

From the authors' responses it is clear that the smallest timescale at which the drift is measured in this study is much larger than that in previous studies, and this may be a main reason why some results are in apparent contradiction to those from other studies (for example that the effects don't seem to "accumulate" over time). The analogy of random-walk on a sphere is helpful and is also compatible with some previous studies. In the new text in the Discussion, an explanation is provided in the paragraph starting at line 577. It would be more beneficial to the reader to emphasize this distinction more clearly (i.e. that the change in the response vector is being measured in the "stabilized" regime). I suggest to clarify this also in the Introduction or when the results are presented, as this underlies many findings in the study.

We agree, an earlier emphasis on this distinction would be helpful. **We have added footnote 4 to qualify these results in the context of the time scales we analyze.**

Did the authors find any evidence of a short-term/within-session drift from analyses other than SVC? If short-term drift cannot be reliably verified or measured, this should be mentioned in the paper. Please also compare to the study of Ref [11] in the same data that found within minutes drift. Along these lines, please also mention in the main text (e.g. in the Discussion) that the variational space may contain, to some extent, the within session drift, as was explained in response to my third question.

We believe the short-term/within-session drift is indeed verified by the SVC, but since the primary goal of this work was to investigate properties of longer time scale drift (and hence our analysis methods relied on several repeats of the same stimulus) we did not pursue an analysis of within-session drift further. Indeed, we mention similarities to the long timescale analysis to that of [11] throughout. **We added two sentences in the discussion about the variational space containing within-session drift, with specific reference to the findings of [11]**.
